# Five New Amicoumacins Isolated from a Marine-Derived Bacterium *Bacillus subtilis*

**DOI:** 10.3390/md10020319

**Published:** 2012-02-03

**Authors:** Yongxin Li, Ying Xu, Lingli Liu, Zhuang Han, Pok Yui Lai, Xiangrong Guo, Xixiang Zhang, Wenhan Lin, Pei-Yuan Qian

**Affiliations:** 1 KAUST Global Collaborative Research, Division of Life Science, Hong Kong University of Science and Technology, Clear Water Bay, Hong Kong, China; Email: liyongxin@ust.hk (Y.L.); boxuying@ust.hk (Y.X.); leonie@ust.hk (L.L.); zhuanghan@ust.hk (Z.H.); cycylai@ust.hk (P.Y.L.); 2 Advanced Nano-fabrication, Imaging & Characterization Core lab, King Abdullah University of Science and Technology, Thuwal 23955-6900, Saudi Arabia; Email: xianrong.guo@kaust.edu.sa (X.G.); xixiang.zhang@kaust.edu.sa (X.Z.); 3 State Key Laboratory of Natural and Biomimetic Drugs, Peking University, Beijing 100191, China; Email: whlin@bjmu.edu.cn

**Keywords:** antibacterial, cytotoxicity, lipoamicoumacin, *Bacillus subtilis*

## Abstract

Four novel amicoumacins, namely lipoamicoumacins A–D (**1**–**4**), and one new bacilosarcin analog (**5**) were isolated from culture broth of a marine-derived bacterium *Bacillus subtilis*, together with six known amicoumacins. Their structures were elucidated on the basis of extensive spectroscopic (2D NNR, IR, CD and MS) analysis and in comparison with data in literature.

## 1. Introduction

Highly diverse marine microbes not only act as a driving force of material cycles in the marine ecosystems but also serve as an excellent source of novel compounds with great potential for biomedical and ecological applications [1,2]. However, natural product discoveries from marine microbes in the Red Sea remain largely unexplored. Accordingly, we have recently initiated a program to discover bioactive natural products from microorganisms isolated from some under-explored ecological niches in the Red Sea. Based on the cytotoxicity and antibacterial activities of the crude extract of its fermented broth, strain B1779, identified as *Bacillus subtilis* based on 16s DNA gene sequence, was chosen for further chemical investigation. Previous chemical studies of the Genus *Bacillus* have led to the isolation and identification of six isocoumarin type metabolites [3]. Some of these exhibit different biological activities, such as cytotoxic [4,5], antibacterial [5,6], antiulcer [7,8,9] and plant-growth inhibitory activities [10]. Our ongoing chemical investigation of *B. subtilis* strain B1779 resulted in the isolation of 11 amicoumacins, including four novel lipoamicoumacins that were designated as lipoamicoumacins A–D (**1**–**4**), one new bacisarcin C (**5**) and six known analogues (**6**–**11**). Compounds **6**–**7** showed significant cytotoxic activity against HeLa cells and strong antibacterial activities against *Staphylococcus aureus* and *Loktanella hongkongensi*. In this paper, we report the fermentation, isolation, structures elucidation, and cytotoxic and antibacterial activities of isolated compounds from a *Bacillus subtilis* strain B1779. 

## 2. Results and Discussion

Strain *Bacillus subtitlis* B1779 was cultivated in a sea salt-based medium for 4 days with vigorous shaking (160 rpm). The UPLC-MS and bioassay guided fractionation revealed that the ethyl acetate (EtOAc) extract of its fermentation broth contained bioactive secondary metabolites with molecular weights ranging from *m/z*: 390 to 730. Moreover, analysis of the ^1^H NMR and UV spectra of this extract revealed that the bioactive fractions were mainly comprised of isocoumacin-type compounds with characteristic spectral features and UV absorptions similar to those of amicoumcin A (**7**). Further separation with semi-preparative HPLC resulted in the isolation of 11 constituents with similar UV and NMR features, including five new compounds (**1**–**5**); their structures were determined on the basis of spectroscopic analysis and upon comparison with data in the literature. Compounds **1**–**4** were identified as novel lipo-amicoumacins, which are referred to herein as Lipoamicoumacin A–D (**1**–**4**).

### 2.1. Structure Elucidation of New Amicoumacins

**Figure 1:**
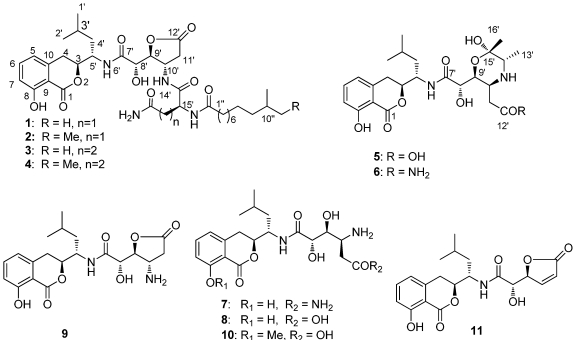
Amicoumacins (**1**–**11**) isolated from *Bacillus subtilis* strain B1779.

**Figure 2:**
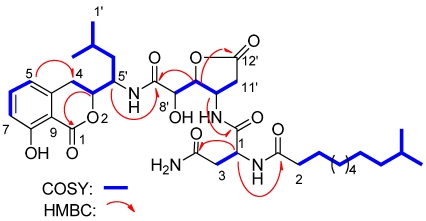
Select HMBC and COSY correlations of lipoamicoumacin A (**1**).

**Figure 3:**
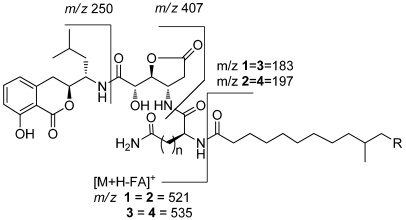
ESI-MS fragmentations of lipoamicoumacins A–D (**1**–**4**).

Lipoamicoumacin A (**1**) was obtained as a white amorphous solid. Based on HRESIMS (*m/z* 703.3921 [M + H]^+^) data we established its molecular formula as C_36_H_54_N_4_O_10_, indicating twelve degrees of unsaturation. The IR absorptions at 3432, 1667 and 1545 cm^−1^ in association with the ^13^C NMR spectroscopic data suggested the presence of hydroxyl, carbonyl and aromatic groups. The ^1^H NMR spectra exhibited three aromatic proton singlets for a tri-substituted aromatic unit at δ_H_ 7.47 (1H, dd, H-6), 6.85 (1H, d, H-7) and 6.81 (1H, d, H-5), three oxymethines at δ_H_ 4.79 (1H, t, H-9'), 4.68 (1H, dd, H-3) and 4.42 (1H, d, H-8'), three nitrogen-bond methines at δ_H_ 4.51 (1H, dt, H-10'), 4.65 (1H, dd, H-15') and 4.29 (1H, m, H-5'), a broad peak at δ_H_ 1.30 (10H, brs, H4''~8'') and four methyl resonances at δ_H_ 0.97 (3H, d, H-2'), 0.89 (3H, d, H-1') and 0.88 (6H, d, H-11'', H-12''). The ^13^C NMR spectra provided five ester/amide carbonyl resonances at δ_C_ (171.2–178.1), six aromatic carbons for a trisubstituted aromatic ring, three oxymethines and three nitrogen-bonded sp^3^ carbons. In addition, the UV spectrum was nearly identical to that reported for amicoumacins (λ_max_ 206, 247 and 314 nm) [8], suggesting that **1** possessed a similar dihydroisocoumarin chromophore. The gross structure of **1** was further established by analyses of the ^1^H, ^13^C, ^1^H–^1^H COSY, HMQC and HMBC NMR spectral data (Figure 1 and Figure 2), indicating an amicoumacin unit was linked to a fatty acid chain. The NMR spectroscopic data of **1** were highly similar to those from the combination of amicoumacin C (**9**) and lipoamide B [8,11] with the exceptions of the Δδ = 1.6 ppm upfield shift of C-10' (δ_C_ 48.6) and Δδ = 0.23 ppm downfield shift of H-10' (δ_H_ 4.51). The differences were due to the linkage of the lipoamide group at C-10' in compound **1** when comparing it with **9**. These assignments were also supported by HMBC correlations from H-10' to C-14' and ESI-MS fragmentations (Figure 3). The ESI-MS spectrum showed fragment ions corresponding to the loss of an acyl group from the *iso* C_12_ fatty acid side strain (*m/z* 521) and sequential loss of asparagine (Asn) (*m/z* 407) and dihydroisocoumarin fragment ion (*m/z* 250). The relative configuration of **1** was determined by NOE correlations of H-3/H-4' and H-5'/H-8', which shares similar stereochemical configurations with amicoumacin C (**9**). To further confirm the absolute configuration of the dihydroisocoumarin unit, the CD spectrum of **1** and **9** was compared. The CD spectrum of **1** showed positive Cotton effects at 221 and 240 nm and negative Cotton effects at 260 and 310 nm, which were in good agreement with those for compound **8** and **9** [9,10]. Thus, the structure of **1** was determined as illustrated in Figure 1. 

The molecular formula of lipoamicoumacin B (**2**) was established as C_37_H_56_N_4_O_10_, based on the HRESIMS (*m/z* 717.4062 [M + H]^+^, calcd. 717.4069), which was 14 amu higher than that of **1**. The NMR spectroscopic data of **2** (Supporting Information, Table 1) indicated that its structure was closely related to that of **1**. The only difference was the presence of an additional methyl group (δ_H_ 0.86, δ_C_ 11.9) in **2**, located at C-11''. HMBC correlation between H-12'' and C-10'' and COSY correlations from H-12'' to H-11'', H-11'' to H-10'' and H-10'' to H-13'' confirmed that the structure of **2** was a C-11'' methylated analog of **1**, seen also with the ESIMS fragmentation analysis. 

**Table 1:** NMR data of lipoamicoumacins A (**1**).

lipoamicoumacins A (1)							
Position	δ_C_, mult	δ_H_, (*J* in Hz)	HMBC	Position	δ_C_, mult	δ_H_, (*J* in Hz)	HMBC
1	171.2, C			11'	36.7, CH_2_	2.45, dd (2.6, 18.0), 3.01 m	12'
3	82.7, CH	4.68, m	1	12'	178.1, C		
4	30.8, CH_2_	2.98, dd (3.5, 16.0), 3.06, m	5, 9, 10			Asparagine	
5	119.8, CH	6.81, d (7.6)	4, 7, 9	14'	173.3, C		
6	137.8, CH	7.47, dd (7.5, 8.4)	8, 10	15'	51.3, CH	4.65, dd (6.5, 7.0)	14', 1''
7	116.9, CH	6.85, d (8.4)	5, 8, 9	16'	38.2, CH_2_	2.57, dd (7.1, 15.2), 2.67, dd (6.4,15.2)	14', 17'
8	163.3, C			17'	174.8, C		
9	109.6, C			18'			
10	141.4, C					Fatty acid	
1'	22.1, CH_3_	0.89, d (6.6)	2', 4'	1''	176.3, C		
2'	24.0, CH_3_	0.97, d (6.6)	1', 4'	2''	37.0, CH_2_	2.24, t (8.0)	1''
3'	26.0, CH_3_	1.68, m		3''	27.0, CH_2_	1.60, m	
4'	40.4, CH_2_	1.43, m, 1.82, m		4''~8''	30.5–31.2	1.30, brs	
5'	50.7, CH	4.29, m	7'	9''	38.3, CH_2_	1.17 m, 1.30 m	
7'	172.8, C			10''	27.0, CH	1.53 m	
8'	73.6, CH	4.42, d (2.5)	7', 10'	11''	23.2, CH_3_	0.88, t (6.8)	9''
9'	87.9, CH	4.79, t (2.4)	7', 12'	12''	23.2, CH_3_	0.88, t (6.8)	9''
10'	48.6, CH	4.51, dt (9.1, 2.0)	14'				

Lipoamicoumacin C (**3**) had a molecular formula of C_37_H_56_N_4_O_10_, which was established by the HRESIMS data (*m/z* 717.4062 [M + H]^+^, calcd. 717.4069). The NMR data of **3** (Supporting Information, Table 1) were comparable to those of **1**, with the exception of an additional methylene at δ_H_ 2.25 (2H, t, H-17') and the Δδ = 3.1 ppm downfield shift of C-18' (δ_C_ 177.8). Analysis of ESIMS fragmentation ions (Figure 3) revealed the presence of a glutamine group (∆*m/z* 535–407) in **3**, instead of an asparagine group (∆*m/z* 521–407) in **1**. The suggestion was further confirmed by HMBC correlations of H-10'/C-14', H-15'/C-1'' and ^1^H–^1^H COSY correlations of H-17'/H-16' and H-16'/H-15'.

Lipoamicoumacin D (**4**) had a molecular formula of C_38_H_58_N_4_O_10_, which was established from the HRESIMS (*m/z* 731.4215 [M + H]^+^, calcd. 731.4222). The molecular weight of **4** was also 14 amu higher than that of **3**. The NMR and MS spectroscopic data of **4** indicated that its structure is nearly identical to that of **3**, with the exception of an additional methyl group located at C-11'' in **4**. 

Bacilosarcin C (**5**) had a molecular formula of C_24_H_34_N_2_O_9_, which was established from the HRESIMS (*m/z* 495.2344 [M + H]^+^, calcd. 495.2337). The ^1^H NMR spectrum exhibited three aromatic proton singlets for one aromatic spin system at δ_H_ 7.46 (1H, dd, H-6), 6.85 (1H, d, H-7) and 6.82 (1H, d, H-5), three oxymethines at δ_H_ 4.68 (1H, dd, H-9'), 4.66 (1H, dd, H-3) and 4.14 (1H, d, H-8'), two nitrogen-bonded methines at δ_H_ 4.34 (1H, dt, H-5'), 3.91 (1H, m, H-10') and 3.43 (1H, q, H-14'), two methylenes at δ_H_ 3.10 (1H, dd, H-4) and 2.96 (1H, dd, H-4), δ_H_ 3.06 (1H, dd, H-11') and 2.92 (1H, dd, H-11'), and four methyl resonances at δ_H_ 1.28 (3H, d, H-13'), 1.26 (3H, s, H-16'), 0.98 (3H, d, H-2') and 0.94 (3H, d, H-1'). The UV absorptions at λ_max_ 206, 247 and 314 nm in association with the NMR data suggested that compound **5** also possesses an amicoumacin unit. The NMR data of **5** (Supporting Information, Table 2) were mostly the same as bacilosarcin B (**6**) [10], except for the Δδ = 0.20 ppm downfield shift of H-11' (δ_H_ 3.06). The difference was due to the linkage of a hydroxyl group (**5**) as opposed to an amide group (**6**) at C-12'. These assignments were also supported by molecular composition and a HRESI-MS fragment ion (*m/z*: 477.2238 [M − H_2_O + H]^+^) corresponding to the intermolecular dehydration between C-9' and C-12'. The relative configurations of **5** were determined by the NOE correlations of H-4'/H-9', H-12'/H-8' and H-12'/14', which were in good agreement with those for compound **6**. The stereochemistry of **5** was assigned to be the same as that of **6** based on their similar Cotton effects observed in the CD spectrum and similar levo specific rotation.

Based on spectroscopic analyses and the comparison of their spectroscopic data with those reported in the literature, six known compounds were identified as bacilosarcin B (**6**) [10], amicoumacin A–C (**7**–**9**) [6,9], 6-[[1-(3,4-dihydro-8-methoxy-l-oxo-1H-2-benzopyran-3-yl)-3-methylbutyl]amino]-4,5-dihydroxy-6-oxo-3-ammoniohexanoate (**10**) and AI-77-F (**11**) [8].

### 2.2. Biological Evaluation

All isolated compounds were evaluated for their cytotoxicity against human cervical carcinoma epithelioid cell line HeLa using MTT methods, and for their antibacterial activities against *Staphylococcus aureus*, *Bacillus subtilis*, *Loktanella hongkongensis*, *Peseudomonas aeruginosa* and *Chromobacterium violaceum* by MIC assay .Only compounds amicoumacin A (**7**) and bacilosarcin B (**6**), which have an amide functional group at C-12', exhibited cytotoxicity against the HeLa cells with IC_50_ values of 33.60 and 4.32 μM respectively, indicating that the C-12' amide group of amicoumcin plays a critical role in cytotoxicity. This was further supported by comparision of cytotoxicity between compounds **7** and **8** and between compounds **5** and **6**. Compound **7**, with an IC_50_ value of 4.32 μM showed 30-fold higher cytotoxicity than **8**. Previous research also reported that C-12 amide groups of amicoumacin played a critical role in antibacterial activities against methicillin-resistant Staphylococcus aureus (MRSA) [12], which was based on the comparison of amicoumacin A (**7**) and amicoumacin B (**8**). Herein, we studied the antibacterial structure-activity relationships based on 11 amicoumacins against three target bacterial strains. Our results indicate that only those compounds with C-12' amide groups exhibited antibacterial activities against *B. subtilis*, *S. aureus* and *L. hongkongensis* (Supporting Information, Table 3), which strongly supported that the C-12' amide group of amicoumcin acts as a pharmacophore in antibacterial activities. This conclusion was further supported by comparing antibacterial activities between compounds **7** and **8** and between compound **5** and **6**. Compound **7** exhibited antibacterial activities against *B. subtilis*, *S*. *aureus* and *L. hongkongensis*, which were about six-fold higher than those of compound **8**. 

While microbial secondary metabolites have been extensively used as antimicrobial or antitumor therapeutic agents, the biological functions of the metabolites in microbes that produce them have only been studied in a few cases. For instance, xenocoumacin**-**1, the major antimicrobial compound produced by *Xenorhabdus nematophila*, was shown to suppress their competitors of the host and subsequently convert xenocoumacin**-2**, which has a much weaker antimicrobial activity, to avoid self-toxicity [13]. In *Bacillus* genus, surfactins were produced to serve as signaling molecules to activate the pathway related to biofilm information [14], while sporulenes was shown to serve as a chemical barrier against oxidative stress [15]. However, the biological role of amicoumacins in the life cycle of *B. subtillis* is far from being understood. It will therefore be intriguing to investigate the chemical ecology of amicoumacins in the future.

## 3. Experimental Section

### 3.1. General Experimental Procedures

Optical rotations were recorded on an Autopol III Rudolph research automatic polarimeter, whereas IR spectra were obtained on a Thermo Nicolet Nexus 470 FT-IR spectrometer. NMR spectra were measured on a Bruker Avance-500/700 FT 500/700 MHz NMR spectrometer using trimethylsilane (TMS) as an internal standard. CD spectra were recorded on J-810-150s spectropolarimeter (Jasco, Germany). HRESIMS spectra were recorded on a Bruker micrOTOF II ESI-TOF-MS spectrometer. Sephadex LH-20 (18–110 μm) was purchased from Pharmacia Co (Japan), whereas octadecylsilane (ODS) (50 μm) was purchased from YMC Co. (Japan). HPLC was performed on a Waters 600 apparatus using a semi-preparative C-18 Phenomenex Luna 5 μm (10 mm × 250 mm) column and monitored by a UV detector (Waters 2475). 

### 3.2. Bacterial Material and Identification

The *Bacillus subtilis* strain B1779 was isolated from a marine sediment sample which was collected by a multicore sampler at a depth of 1000 m in the Red Sea, in April 2010. This strain was isolated by a standard dilution-plating technique. Bacteria in the sediment were released by vigorous shaking in autoclaved filtered seawater and inoculated in a serial dilution on 1.5% agar composed of 10 g starch and 2 g yeast extract dissolved in 1 L filtered seawater. According to its 16S rRNA gene sequence (accession number: HM585050.1), this strain belongs to *Bacillus subitlis* group. A voucher strain of this *Bacillus subtilis* strain has been preserved at the Costal Marine Lab, Hong Kong University of Science and Technology. Genomic DNA was extracted from the cells using the TaKaRa MiniBEST Bacterial Genomic DNA Extraction Kit (TaKaRa, China). The 16S rRNA gene of B1779 was amplified by a pair of primers of 8F (5'-AGAGTTTGATCCTGGCTCAG-3') and 1525R (5'-AAGGAGTGWTCCARCC-3') with Vent DNA polymerase (NEB) and sequenced using Applied Biosystems 3100 automated DNA sequencer, as previously described [16,17]. The resulting 16S rRNA gene sequence was compared with sequences obtained from the NCBI nucleotide database using BLAST search [18] to locate approximate phylogenetic affiliation.

### 3.3. Fermentation and Extraction

The bacterium was cultured in multiple 3 L flasks containing SYT culture medium (1% of starch, 0.4% of yeast extract and 0.2% tryptone) in seawater at 25 °C with agitation (160 rpm) for 4 days until the stationary phase was reached. In total, 20 L of fermented broth were obtained. The bacterial culture broth was extracted with an equal volume of ethyl acetate (EtOAc) three times and gave a total of 11 g of crude extract.

### 3.4. Isolation

The crude EtOAc extract (11.0 g) was fractionated by ODS open column chromatography eluting with a step gradient from 40% methanol in water to 100% methanol to give six fractions (F1–F6). UPLC-MS analysis and cytotoxicity bio-assay showed that bioactive isocoumacins were mainly concentrated in fractions 3 and 4 (named as F3 and F4). F3 (0.39 g) was separated by semi-preparative HPLC with a gradient mobile phase (MeCN-H_2_O, from 30 % to 50 %) to obtain **5** (3.6 mg), **6** (4.7 mg), **7** (25.0 mg), **8** (11.5 mg), **9** (18.0 mg), **10** (22 mg) and **11**(6.2 mg), respectively. F4 (0.42 g) was subjected to Sephadex LH-20 and subsequently separated with semi-preparative HPLC eluting with a gradient mobile phase (MeCN-H_2_O, from 60% to 80%) to yield the compounds **1** (1.9 mg), **2** (1.6 mg), **3** (3.5 mg) and **4** (4.8 mg), respectively.

### 3.5. Spectroscopic Data of Compounds

#### 3.5.1. Lipoamicoumacin A (**1**)

White amorphous powder; [α]^27^_D_ −12.0 (*c* 0.20, MeOH); IR (KBr) *ν*_max_ 3432, 3047, 2955, 2925, 2101, 1794, 1667, 1545, 1462, 1371, 1026 and 974 cm^−1^; UV (MeOH) λ_max_ (log *ε*) 206 (4.22), 247 (3.44) and 314 (3.29) nm; for ^1^H and ^13^C NMR data, see Table 1; HREIMS *m/z* 703.3921 [M + H]^+^ (calcd. for C_36_H_55_N_4_O_10_, 703.3913); ESI-MS fragmental ions (*m/z* 521.2, 407.2, 297.2, 250.1). 

#### 3.5.2. Lipoamicoumacin B (**2**)

White amorphous powder;[α]^27^_D_ 4.0 (*c* 0.20, MeOH); IR (KBr) *ν*_max_ 3433, 3304, 2924, 2853, 1790, 1663, 1540, 1461, 1370, 1020 and 873 cm^−1^; UV (MeOH) λ_max_ (log *ε*) 206 (4.18), 247 (3.45) and 314 (3.32) nm; for ^1^H and ^13^C NMR data, see Table 1 in Supporting Information; HREIMS *m/z* 717.4062 [M + H]^+^, (calcd. for C_37_H_57_N_4_O_10_, 717.4069); ESI-MS fragmental ions (*m/z* 521.2, 407.2, 311.2, 250.1).

#### 3.5.3. Lipoamicoumacin C (**3**)

White amorphous powder; [α]^27^_D_ −25.0 (*c* 0.20, MeOH); IR (KBr) *ν*_max_ 3424, 3314, 3068, 2926, 2856, 1793, 1665, 1541, 1463, 1371, 1220, 1166, 1112 and 872 cm^−1^; UV (MeOH) λ_max_ (log *ε*) 206 (4.13), 247 (3.43) and 314 (3.29) nm; for ^1^H and ^13^C NMR data, see Table 1 in Supporting Information 1; HREIMS *m/z* 717.4062 [M + H]^+^ (calcd. for C_37_H_57_N_4_O_10_, 717.4069); ESI-MS fragmental ions (*m/z* 535.3, 407.2, 311.2, 250.1, 183.2).

#### 3.5.4. Lipoamicoumacin D (**4**)

White amorphous powder; [α]^27^_D_ −19.2 (*c* 0.20, MeOH); IR (KBr) *ν*_max_ 3432, 3045, 2924, 2856, 1792, 1664, 1538, 1462, 1368, 1115 and 873 cm^−1^; UV (MeOH) λ_max_ (log *ε*) 206 (4.15), 247 (3.43) and 314 (3.30) nm; for ^1^H and ^13^C NMR data, see Table 1 in Supporting Information 1; HREIMS *m/z* 731.4215 [M + H]^+^, (calcd. for C_38_H_59_N_4_O_10_, 731.4222); ESI-MS fragmental ions (*m/z* 535.3, 407.2, 325.3, 250.1, 197.2).

#### 3.5.5. Bacilosarcin C (**5**)

White amorphous powder; [α]^27^_D_ −18.0 (*c* 0.20, MeOH); IR (KBr)*ν*_max_ 3400, 2959, 2880, 1676, 1620, 1586, 1464, 1388, 1205, 1138, 1040, 966 and 804 cm^−1^; UV (MeOH) λ_max_ (log *ε*) 206 (4.06), 247 (3.35) and 314 (3.23) nm; for ^1^H and ^13^C NMR data, see Table 2 in Supporting Information 1; HREIMS *m/z* 495.2344 [M + H]^+^, (calcd. for C_24_H_35_N_2_O_9_, 495.2337); ESI-MS fragmental ions (*m/z* 477.2, 285.3, 250.1).

### 3.6. Cytotoxicity Assay

Human HeLa cells were used in the assay; 80 μL of 1 × 10^5^/mL cells were planted into 96-microwell plates. Twelve hours later the cells were treated with testing samples at various concentrations and then incubated at 37 °C for 48 hours. Afterward, the supernatant was removed and 20 μL of MTT (2.5 mg mL^−1^) were added to each well. After incubation at 37 °C for 4 hours, 100 μL of dimethyl sulfoxide (DMSO) were added to each well and incubated for an additional 20 min. The absorbance of each well was then measured at 570 nm by a Thermo scientific Multiskan FC multiplate photometer (Waltham, MA, USA).

### 3.7. Antibacterial Assay

The antibacterial activities of compound **1**–**11** were evaluated by MIC assay against *Staphylococcus aureus*, *Bacillus subtilis*, *Loktanella hongkongensis*, *Peseudomonas aeruginosa* and *Chromobacterium violaceum*. Briefly, the bacterial strains were inoculated in YP broth (0.2% yeast extract, 0.1% peptone and 1.7% sea salts) and were incubated at 28 °C for 12 h. The stock solution of samples were prepared at 50 mg/mL in DMSO and further diluted to varying concentrations in 96 well plates that contained the incubated microbial strains. After incubation at 28 °C for 24 hours, the absorbance of each well was then measured at 600 nm by a Thermo scientific Multiskan FC multiplate photometer. 

## 4. Conclusion

Bioassay guided investigation of the Red Sea bacterium *Bacillus subtilis* has resulted in the isolation of four novel lipoamicoumacins, lipoamicoumacin A–D (**1**–**4**), together with one new bacilosarcin C and six known amicoumacins. Study of the structure-activity relationship indicated that the C-12' amide functional groups play a critical role in cytotoxicity and antibacterial activities of these amicoumacins. It is particularly noteworthy to mention that lipoamicoumacin A–D (**1**–**4**) is the first subclass of amicoumacin with a lipoamide side chain to be reported from a natural source.

## Supplementary Files

Supplementary File 1: PDF-Document (PDF, 8487 KB)
