# Supplementary material for: Five New Amicoumacins Isolated from a Marine-Derived Bacterium Bacillus subtilis
Source: Mar Drugs. 2012 Feb 3;10(2):319–28. doi: 10.3390/md10020319 (PMC3296999; doi:10.3390/md10020319)
Supplement: Supplementary File 1: — PDF-Document (PDF, 8487 KB) [file marinedrugs-10-00319-s001.pdf]

Supplementary Materials

Cytotoxicity and antibacterial activities of a group of amicoumacins from a marine-derived bacterium *Bacillus subtilis*

Yongxin Li [1], Ying Xu [1], Lingli Liu [1], Zhuang Han [1], Pok Yui Lai [1], Xiangrong Guo [2], Xixiang Zhang [2], Wenhan Lin [3], Pei-Yuan Qian [1,*]

[1] KAUST Global Collaborative Research, Division of Life Science,
Hong Kong University of Science and Technology, Clear Water Bay, Hong Kong;
E-mail: liyongxin@ust.hk (Y.L.); boxuying@ust.hk (Y.X.); leonie@ust.hk (L.L.); zhuanghan@ust.hk (Z.H.); cycylai@ust.hk (P.Y.L.);

[2] King Abdullah University of Science and Technology, Saudi Arabia;
E-mail: xianrong.guo@kaust.edu.sa (X.G.); xixiang.zhang@kaust.edu.sa (X.Z.)

[3] State Key Laboratory of Natural and Biomimetic Drugs, Peking University,
Beijing 100191, P.R. China; E-mail: whlin@bjmu.edu.cn

* Author to whom correspondence should be addressed; E-Mail: boqianpy@ust.hk;
Tel.: + -852- 2358-7331; Fax: +852- 2358-1559

Supporting information 1

Table-1 NMR data of lipoamicoumacins A–D (**1**–**4**) 2

Table-2 NMR data of bacilosarcin C (5) and bacilosarcin B (6) 3

Table 1. NMR data of lipoamicoumacins A–D (**1**–**4**)

	**1**			**2**		**3**			**4**	
position	δ_C, mult	δ_H, (*J* in Hz)	HMBC	δ_C, mult	δ_H, (*J* in Hz)	δ_C, mult	δ_H, (*J* in Hz)	HMBC	δ_C, mult	δ_H, (*J* in Hz)
1	171.2, C			171.2, C		171.2, C			171.2, C	
3	82.7, CH	4.68, m	1	82.7, CH	4.68, m	82.7, CH	4.69, dt (11.0, 3.8)	1	82.7, CH	4.69, dt (11.0, 3.8)
4	30.8, CH_2	2.98, dd (3.5, 16.0), 3.06, m	5, 9, 10	31.0, CH_2	2.98, m, 3.06, m	30.8, CH_2	2.98, dd (3.5, 16.0), 3.06, m	5, 9, 10	30.8, CH_2	2.98, dd (3.5, 16.0) 3.06, m
5	119.8, CH	6.81, d (7.6)	4, 7, 9	119.8, CH	6.81, d (7.4)	119.8, CH	6.81, d (7.5)	4, 7, 9	119.8, CH	6.82, d (7.8)
6	137.8, CH	7.47, dd (7.5, 8.4)	8, 10	137.8, CH	7.47, dd (7.4, 8.6)	137.8, CH	7.47, dd (7.5, 8.4)	8, 10	137.8, CH	7.47, t (7.9)
7	116.9, CH	6.85, d (8.4)	5, 8, 9,	116.9, CH	6.85, d (8.6)	117.0, CH	6.85, d (8.4)	5, 8, 9,	116.9, CH	6.85, d (8.0)
8	163.3, C			163.3, C		163.3, C			163.3, C	
9	109.6, C			109.6, C		109.6, C			109.6 , C	
10	141.4, C			141.4, C		141.5, C			141.4, C	
1'	22.1, CH_3	0.89, d (6.6)	2', 4'	22.1, CH_3	0.89, d (6.6)	22.1, CH_3	0.89, d (6.6)	2', 4'	22.2, CH_3	0.89, d (6.6)
2'	24.0, CH_3	0.97, d (6.6)	1', 4'	23.9, CH_3	0.97, d (6.6)	24.0, CH_3	0.97, d (6.6)	1', 4'	24.0, CH_3	0.97, d (6.6)
3'	26.0, CH	1.68, m		25.9, CH	1.68, m	26.0, CH	1.68, m		25.9, CH	1.68, m
4'	40.4, CH_2	1.43, m, 1.82, m		40.4, CH_2	1.43, m, 1.82, m	40.7, CH_2	1.43, m, 1.82, m		40.7, CH_2	1.44, m, 1.84, m
5'	50.7, CH	4.29, m	7'	50.7, CH	4.29, m	50.7, CH	4.29, m	7'	50.7, CH	4.28, m
7'	172.8, C			172.8, C		172.8, C			172.8, C	
8'	73.6, CH	4.42, d (2.5)	7', 10'	73.7, CH	4.42, d (2.5)	73.6, CH	4.42, d (2.5)	7', 10'	73.6, CH	4.42, d (2.3)
9'	87.9, CH	4.79, t (2.4)	7', 12'	87.9, CH	4.78, t (2.4)	87.9, CH	4.79, t (2.4)	7', 12'	87.8, CH	4.79, t (2.2)
10'	48.6, CH	4.51, dt (9.1, 2.0)	14'	48.6, CH	4.51, m	48.6, CH	4.51, dt (9.1, 2.0)	14'	48.6, CH	4.51, dt (9.2, 2.0)
11'	36.7, CH_2	2.45, dd (2.6, 18.0) , 3.01 m	12'	36.7, CH_2	2.46, dd (2.7, 18.2), 3.01, m	37.0, CH_2	2.42, dd (2.7, 18.2), 3.03, m	12'	37.0, CH_2	2.42, dd (2.6, 18.3), 3.02, m
12'	178.1, C			178.2, C		178.1, C			178.1, C	
Asparagine or Glutamine										
14'	173.3, C			173.3, C		173.9, C			173.8, C	
15'	51.3, CH	4.65,dd (6.5, 7.0)	14', 1''	51.5, CH	4.65, t (6.6)	52.4, CH	4.30, m	14', 1''	52.4, CH	4.30, m
16'	38.2, CH_2	2.57 dd (7.1, 15.2), 2.67, dd (6.4,15.2)	14', 17'	37.9, CH_2	2.57, dd (7.0, 15.4), 2.67, dd (6.4,15.4)	29.2, CH_2	1.86, m, 2.00, m	14', 18'	29.1, CH_2	1.88, m, 2.01, m
17'	174.8, C			174.8, C		32.6, CH_2	2.25, m	18'	32.6, C	2.26, m
18'						177.9, C			177.8, C	

Table 1. *Cont.*

Fatty acid										
1"	176.3, C			176.2, C		176.5, C			176.5, C	
2"	37.0, CH_2	2.24 t (8.0)	1"	37.1, CH_2	2.21 t (8.1)	37.1, CH_2	2.24, t (8.0)	1"	37.0 , CH_2	2.23 t (8.0)
3"	27.0, CH_2	1.60, m		26.8, CH_2	1.59, m	27.0, CH_2	1.60, m		27.0, CH_2	1.60, t
4"~8"	30.5-31.2,			30.5-31.2		30.5-31.2			30.5-31.2,	
9"	38.3, CH_2	1.17 m, 1.30 m		35.8, CH_2	1.30 m	40.4, CH_2	1.18, m, 1.27, m		35.8, CH_2	1.30 m
10"	27.0, CH	1.53 m		28.3, CH	1.29 m	29.5, CH_3	1.53, m		28.3, CH_2	1.29, m
11"	23.2, CH_3	0.88, t (6.8)	9"	37.9, CH_3	1.10 m, 1.30 m	23.2, CH_3	0.88, t (6.8)	9"	37.9, CH_2	1.10 m, 1.30 m
12"	23.2, CH_3	0.88, t (6.8)	9"	11.9, CH_3	0.88, t (6.8)	23.2, CH_3	0.88, t (6.8)	9"	11.9, CH_2	0.88, t (6.8)
13"				19.8, CH_3	0.86, d (6.4)				19.8, CH_3	0.86, d (6.5)

Table 2. NMR data of bacilosarcin C (**5**) and bacilosarcin B (**6**) in CD_3OD

		**5**		**6**			**5**		**6**
position	δ_C mult	δ_H, (*J* in Hz)	HMBC	δ_H, (*J* in Hz)	position	δ_C mult	δ_H, (*J* in Hz)	HMBC	δ_H, (*J* in Hz)
1	171.0 C				4'	40.3, CH_2	1.46, m, 1.80, m	5'	1.46, m, 1.80, m
3	82.5, CH	4.66, dt (3.5, 12.0)	1,	4.67, dt (3.4, 12.0)	5'	50.4, CH	4.34, dt (10.9, 3.9)	7'	4.34, dt (10.9, 3.9)
4	30.8, CH_2	2.96, dd (3.5, 16.5)	3, 5, 9, 10	2.96, dd (3.5, 16.5	7'	173.5, C			
		3.10, dd (16.5, 12.0)	3, 5, 10	3.11, dd (16.5, 12.0	8'	71.9, CH	4.14, d (7.8)	7'	4.11, d (7.9)
5	119.6, CH	6.81, d (7.5)	4, 7, 9	6.81, d (7.5)	9'	68.9, CH	4.68, dd (3.0, 8.0)	7', 11', 15'	4.65, dd (3.1, 8.5)
6	137.5, CH	7.46, dd (7.5, 8.4)	8, 10	7.46, dd (7.5, 8.4)	10'	51.8, CH	3.91, m		3.82, m
7	116.7, CH	6.85, d (8.4)	5, 8, 9,	6.85, d (8.4)	11'	28.8, CH_2	2.92, dd (8.5, 16.5)	12'	2.93, dd (7.5, 18.0)
8	163.1, C						3.06, dd (16.5, 4.0)	12'	2.86, dd (17.5, 3.5)
9	109.3, C				12'	174.4, C			
10	141.2, C				13'	13.9, CH_3	1.28, d (6.5)	15'	1.28, d (6.5)
1'	21.7, CH_3	0.94, d (6.5)	2', 4'	0.94, d (6.5	14'	53.0, CH	3.43, q (6.4)	10', 15'	3.47, q (6.5)
2'	23.7, CH_3	0.98, d (6.5)	1', 4'	0.98, d (6.5	15'	96.2, C			
3'	25.9, CH_2	1.71, m		1.69, m	16'	24.8, CH_3	1.26 s	14'	

Table 3. Antibacterial activities and cytotoxicity of amicoumacin derivates

compound	Antibacterial (MIC, μM)			Cytotoxicity (IC_{50}, μM)
	Bacllus subtillis	*Staphylococcus aureus*	*Loktanella hongkongensis*	Hela
**1**	[a]NA	NA	NA	NA
**2**	NA	NA	NA	NA
**3**	NA	NA	NA	NA
**4**	NA	NA	NA	NA
**5**	NA	NA	NA	NA
**6**	NA	4.05	16.19	33.60
**7**	18.87	18.87	1.18	4.32
**8**	NA	NA	NA	NA
**9**	NA	NA	NA	NA
**10**	NA	NA	NA	NA
**11**	NA	NA	NA	NA
Penicilin G	0.29	0.73	5.81	
cis-DDP				15.87

[a]NA: MIC> 100μM (antibacterial) or IC_{50}>100μM (cytotoxicity)

Cytotoxicity and antibacterial activities of a group of amicoumacins from a marine-derived bacterium *Bacillus subtilis*

Yongxin Li[1], Ying Xu[1], Lingli Liu[1], Zhuang Han[1], Pok Yui Lai[1], Xiangrong Guo[2], Xixiang Zhang[2], Wenhan Lin[3], Pei-Yuan Qian[1]*

[1] KAUST Global Collaborative Research, Division of Life Science, Hong Kong University of Science and Technology, Clear Water Bay, Hong Kong; E-mail: liyongxin@ust.hk (Y. L.); boxuying@ust.hk (Y. X.); leonie@ust.hk (L. L.); zhuanghan@ust.hk (Z. H.); cycylai@ust.hk (P. Y. L.);

[2] King Abdullah University of Science and Technology, Saudi Arabia; E-mail: xianrong.guo@kaust.edu.sa (X. G.); xixiang.zhang@kaust.edu.sa (X. Z.)

[3] State Key Laboratory of Natural and Biomimetic Drugs, Peking University, Beijing 100191, P.R. China; E-mail: whlin@bjmu.edu.cn

* Author to whom correspondence should be addressed; E-Mail: boqianpy@ust.hk; Tel.: + -852- 2358-7331; Fax: +852- 2358-1559

Fig 1-1 ^{1}H NMR spectrum of **1**....4
Fig 1-2 DEPT spectrum of **1**....4
Fig 1-3 COSY spectrum of **1**....5
Fig 1-4 HMQC spectrum of **1**....5
Fig 1-5 HMBC spectrum of **1**....6
Fig 1-6 NOESY spectrum of **1**....6
Fig 1-7 CD spectrum of **1**....7
Fig 1-8 IR spectrum of **1**....7
Fig 1-9 HRESIMS spectrum of **1**....8
Fig 1-10 ESIMS spectrum of **1**....8
Fig 2-1 ^{1}H NMR spectrum of **2**....9
Fig 2-2 DEPT spectrum of **2**....9
Fig 2-3 COSY spectrum of **2**....10
Fig 2-4 HMQC spectrum of **2**....10
Fig 2-5 HMBC spectrum of **2**....11
Fig 2-6 NOESY spectrum of **2**....11
Fig 2-7 CD spectrum of **2**....12
Fig 2-8 IR spectrum of **2**....12
Fig 2-9 HRESIMS spectrum of **2**....13
Fig 2-10 ESIMS spectrum of **2**....13
Fig 3-1 ^{1}H NMR spectrum of **3**....14
Fig 3-2 DEPT spectrum of **3**....14
Fig 3-3 COSY spectrum of **3**....15
Fig 3-4 HMQC spectrum of **3**....15
Fig 3-5 HMBC spectrum of **3**....16
Fig 3-6 IR spectrum of 3....16
Fig 3-7 CD spectrum of **3**....17
Fig 3-8 HRESIMS spectrum of **3**....17
Fig 3-9 ESIMS spectrum of **3**....17
Fig 4-1 ^{1}H NMR spectrum of **4**....18
Fig 4-2 DEPT spectrum of **4**....18
Fig 4-3 COSY spectrum of **4**....19
Fig 4-4 HMQC spectrum of **4**....19
Fig 4-5 HMBC spectrum of **4**....20
Fig 4-6 NOESY spectrum of **4**....20
Fig 4-7 CD spectrum of **4**....21
Fig 4-8 IR spectrum of **4**....21
Fig 4-9 HRESIMS spectrum of **4**....22
Fig 4-10 ESIMS spectrum of **4**....22
Fig 5-1 ^{1}H NMR spectrum of **5**....23
Fig 5-2 ^{1}H NMR spectrum of **6**....23
Fig 5-3 COSY spectrum of **5**....24
Fig 5-4 HMQC spectrum of **5**....24
Fig 5-5 HMBC spectrum of **5**....25

Fig 5-6 NOESY spectrum of **5** 25
Fig 5-7 CD spectrum of **5** 26
Fig 5-8 IR spectrum of **5** 26
Fig 5-9 HRESIMS spectrum of **5** 27

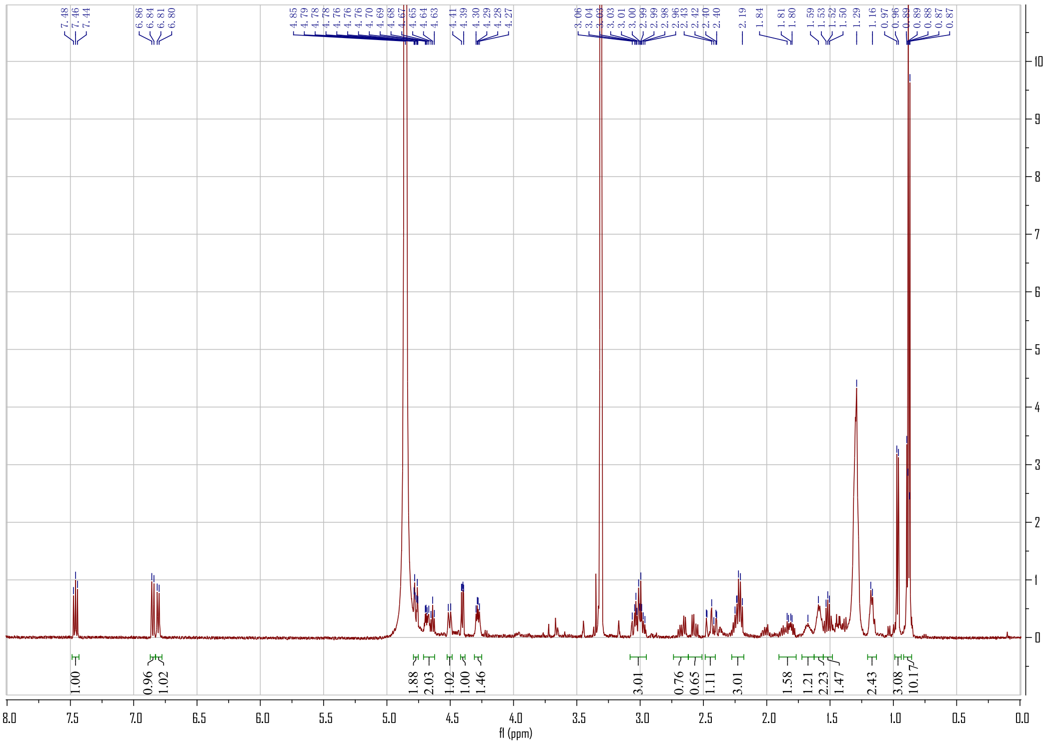

Fig 1-1 1H NMR spectrum of 1

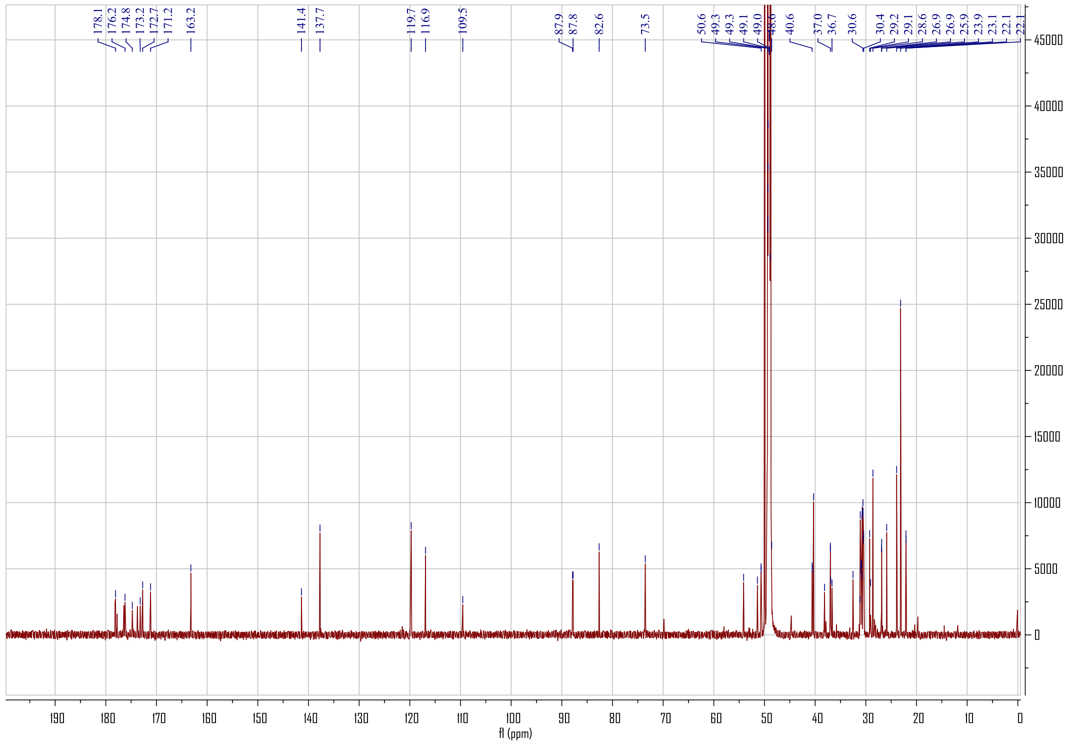

Fig 1-2 DEPT spectrum of **1**

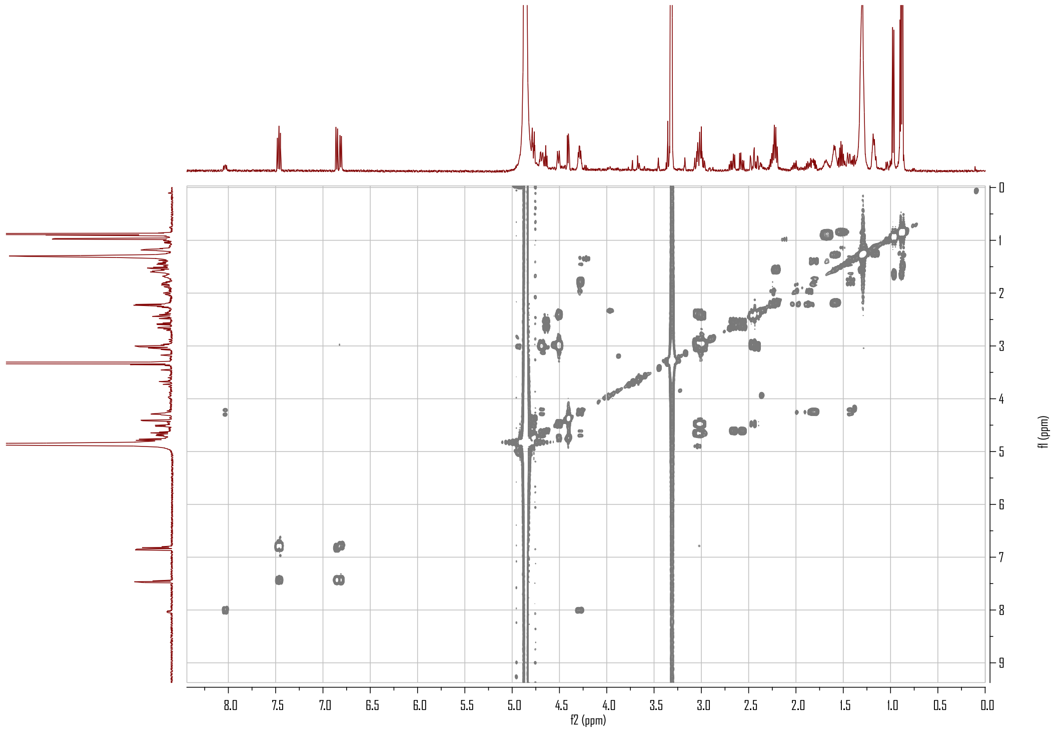

Fig 1-3 COSY spectrum of **1**

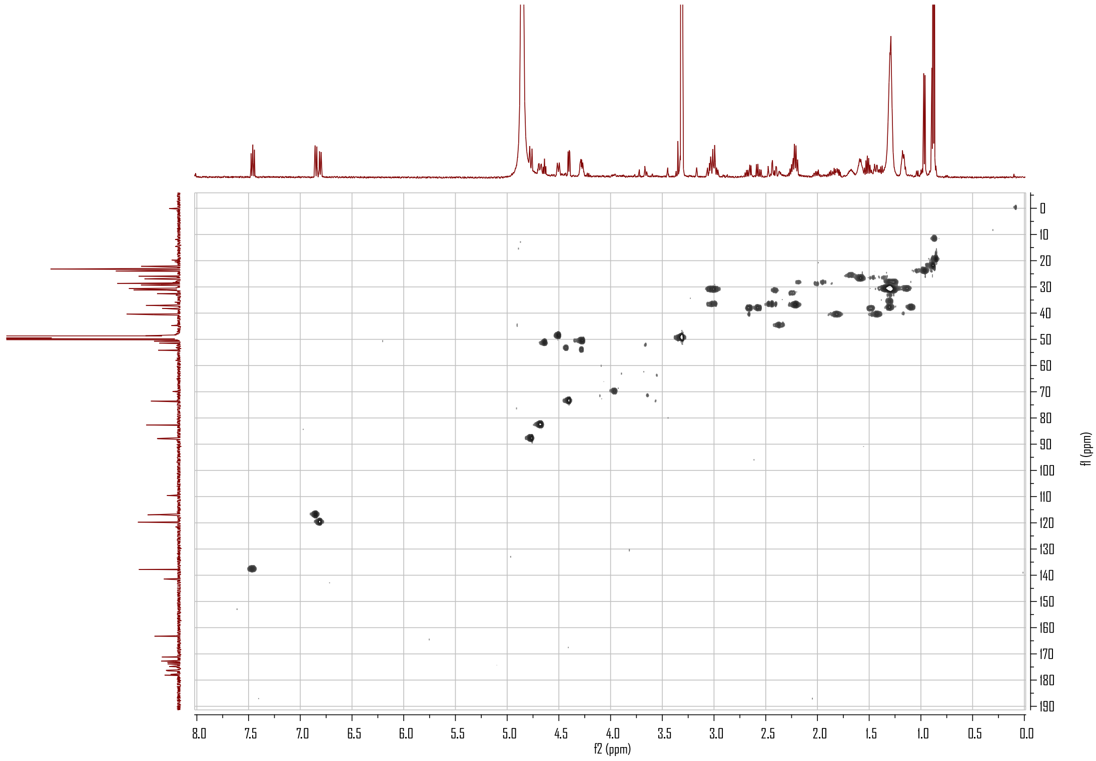

Fig 1-4 HMQC spectrum of **1**

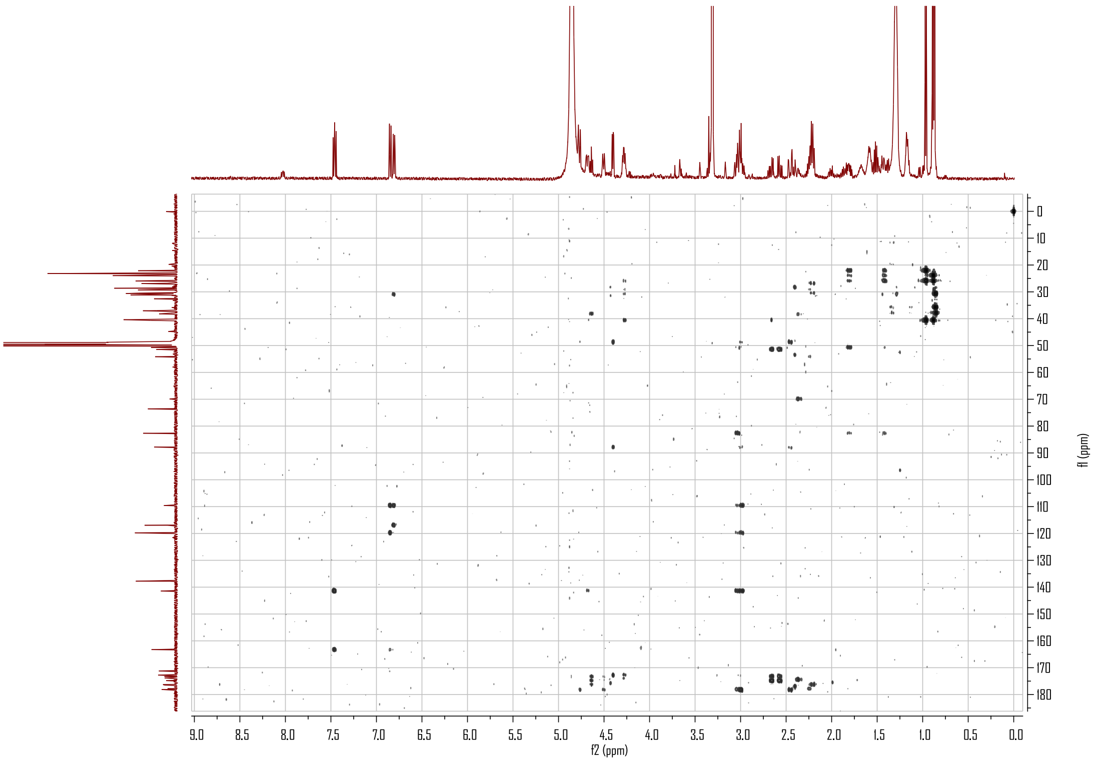

Fig 1-5 HMBC spectrum of **1**

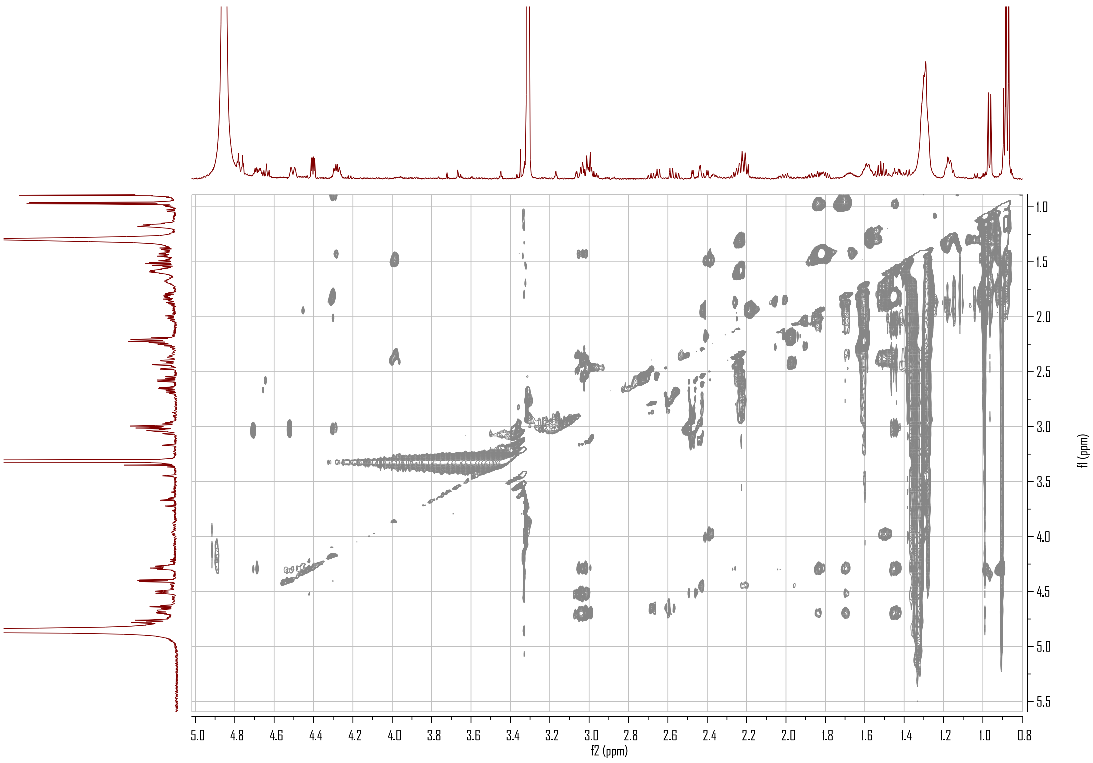

Fig 1-6 NOESY spectrum of **1**

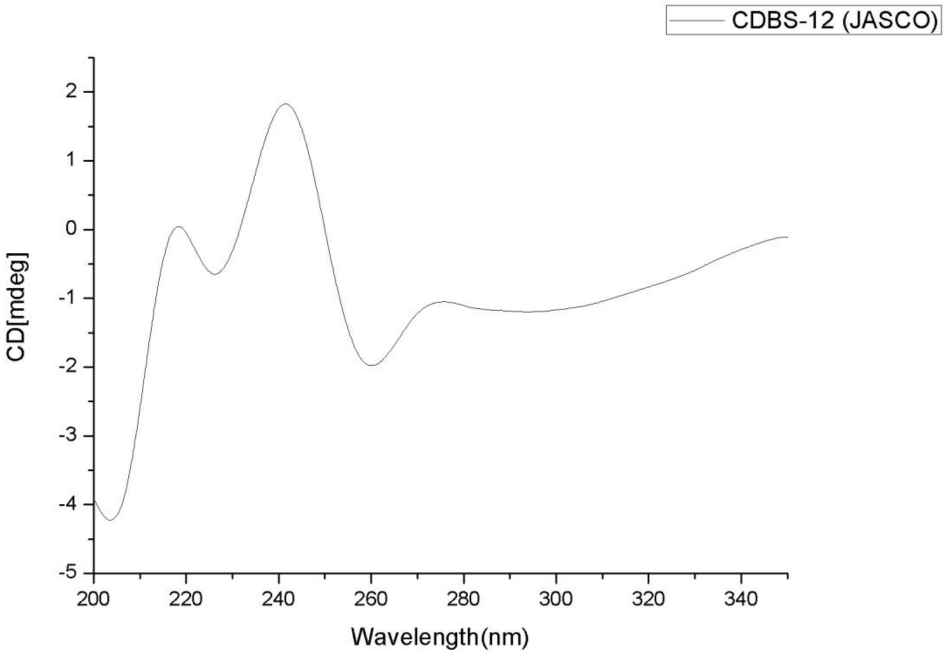

Fig 1-7 CD spectrum of **1**

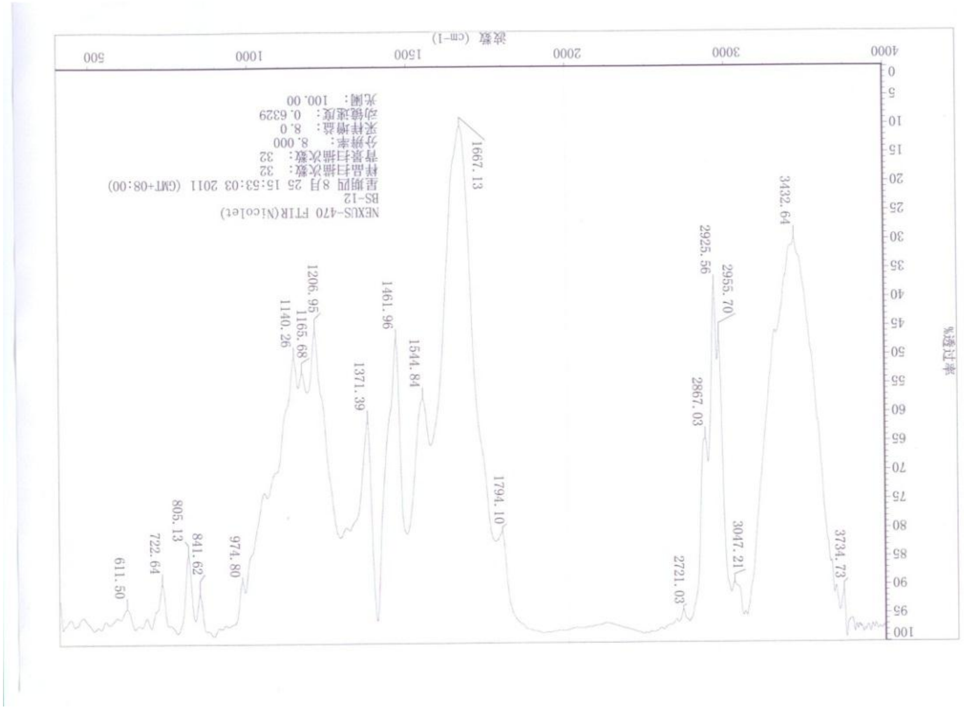

Fig 1-8 CD spectrum of **1**

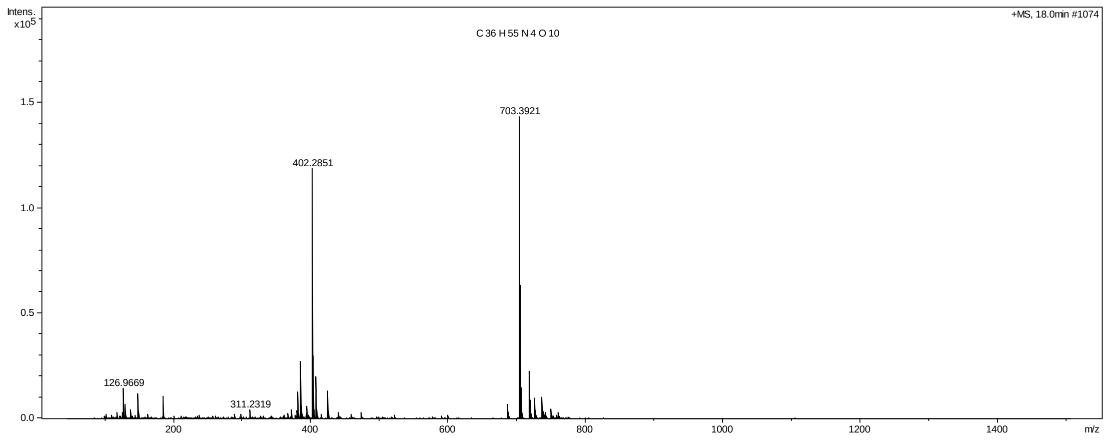

Fig 1-9 HRESIMS spectrum of **1**

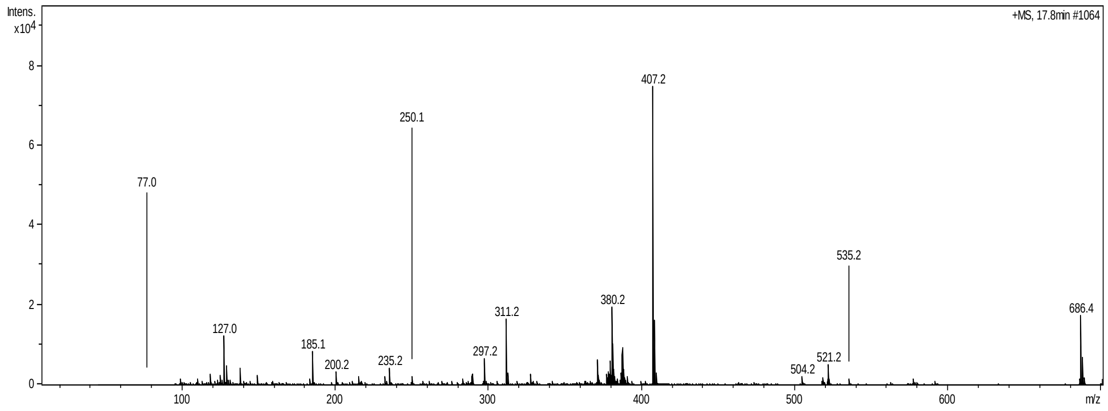

Fig 1-10 ESIMS spectrum of **1**

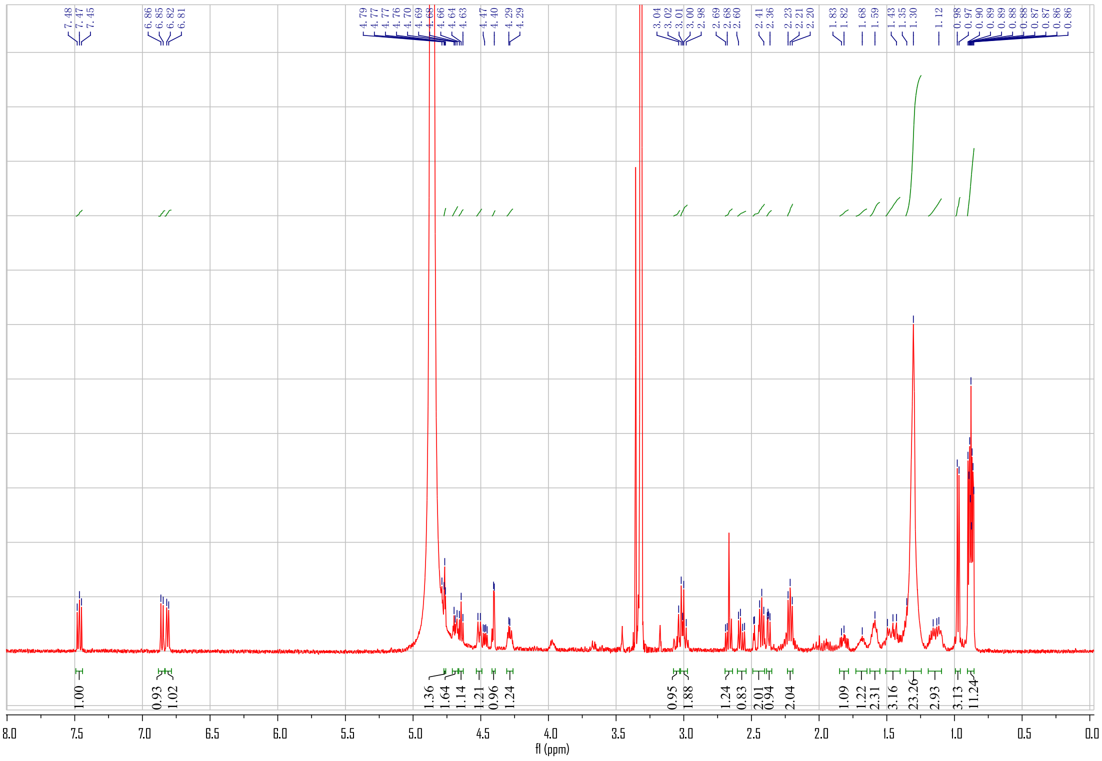

Fig 2-1 ^{1}H NMR spectrum of **2**

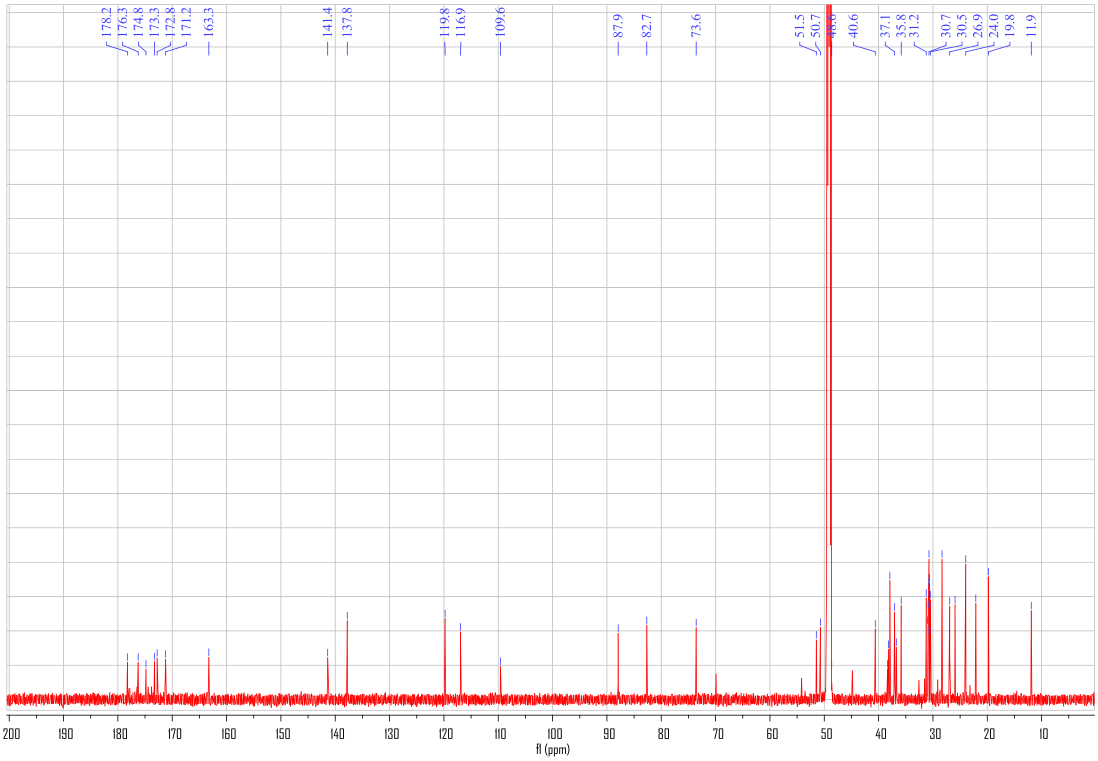

Fig 2-2 DEPT spectrum of **2**

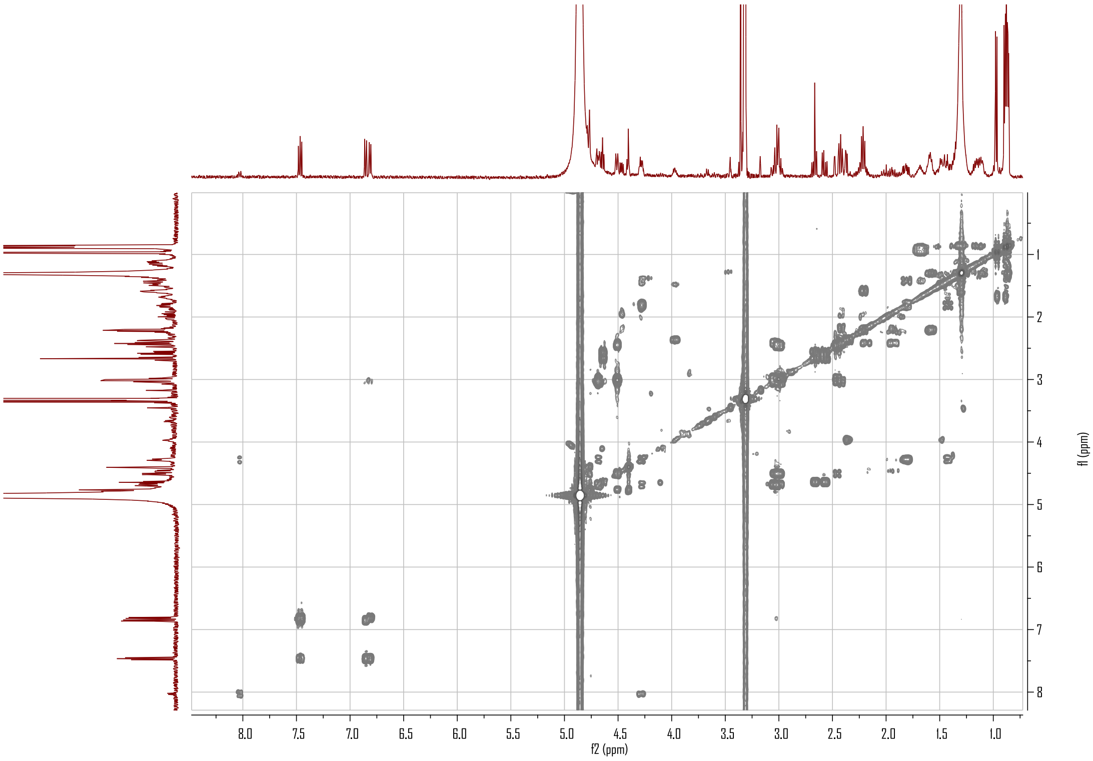

Fig 2-3 ^{1}H-^{1}HCOSY spectrum of **2**

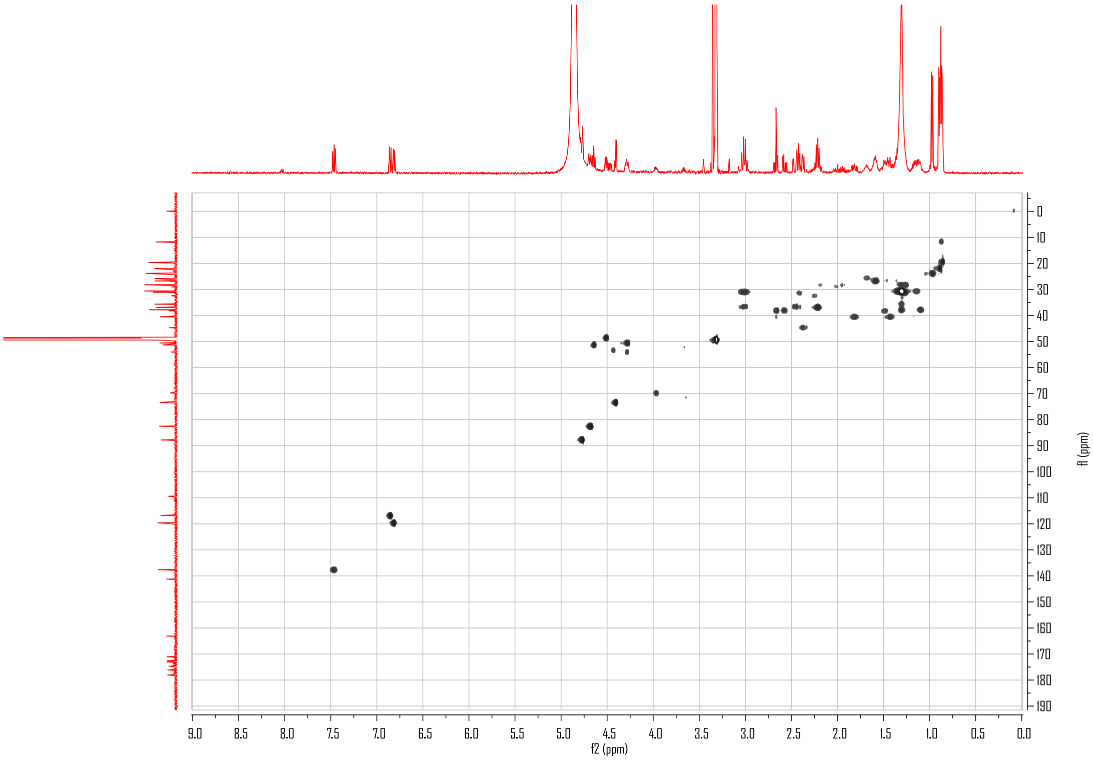

Fig 2-4 HMQC spectrum of **2**

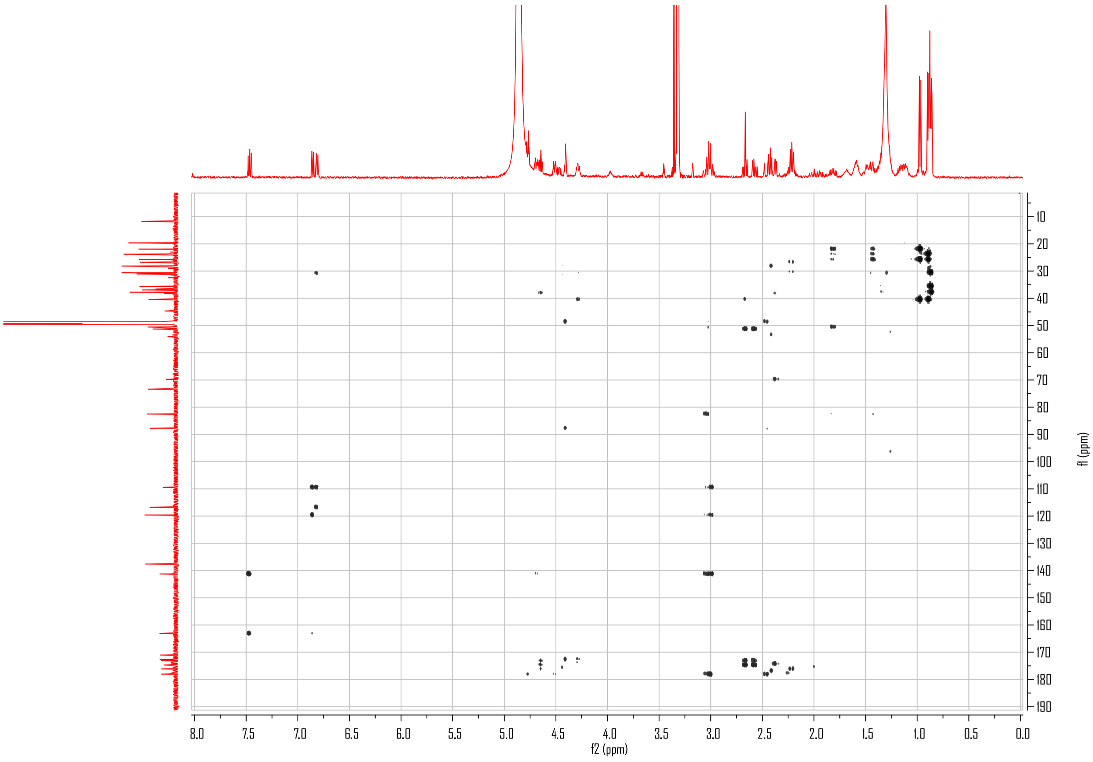

Fig 2-5 HMBC spectrum of **2**

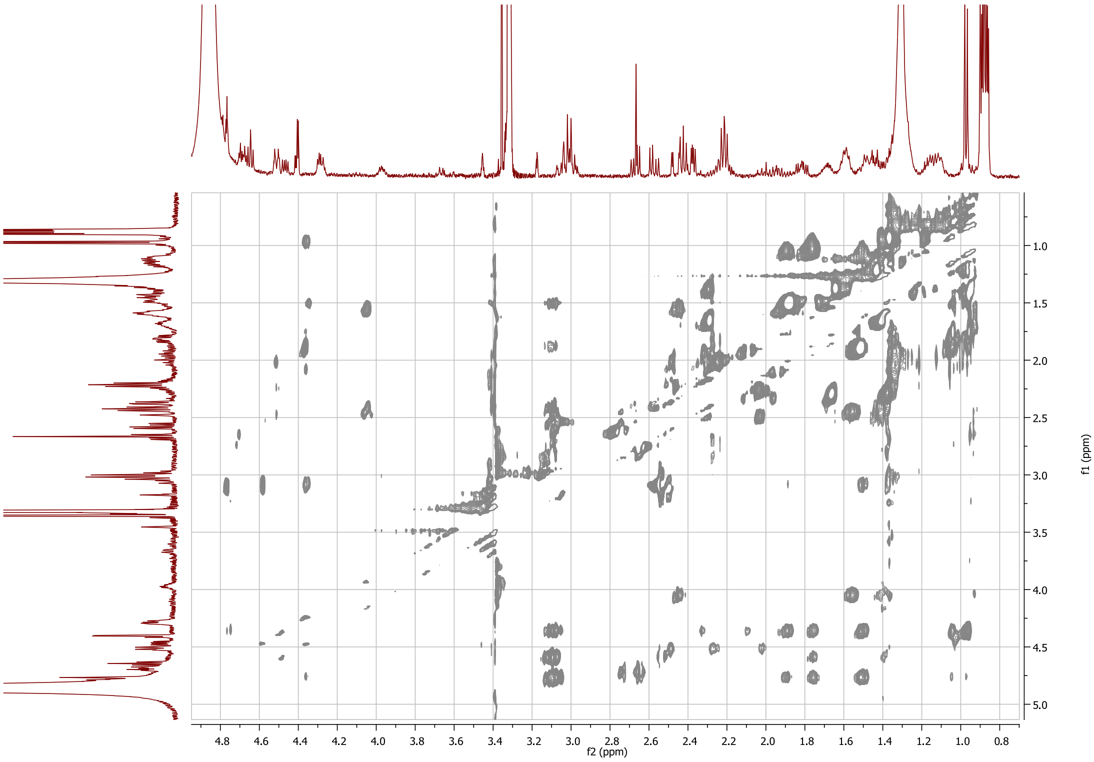

Fig 2-6 NOESY spectrum of **2**

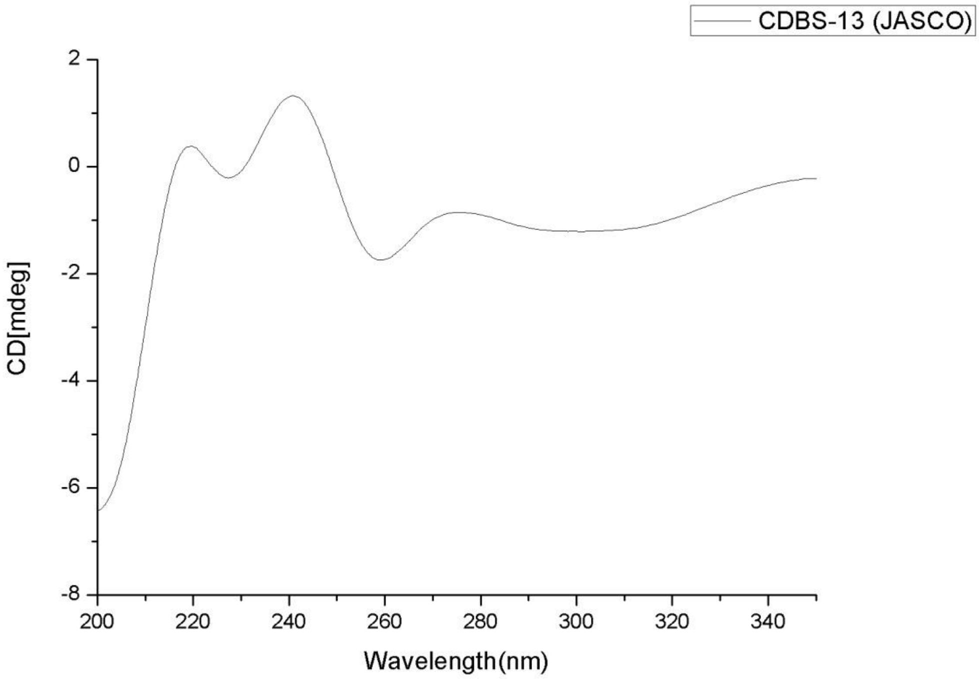

Fig 2-7 CD spectrum of **2**

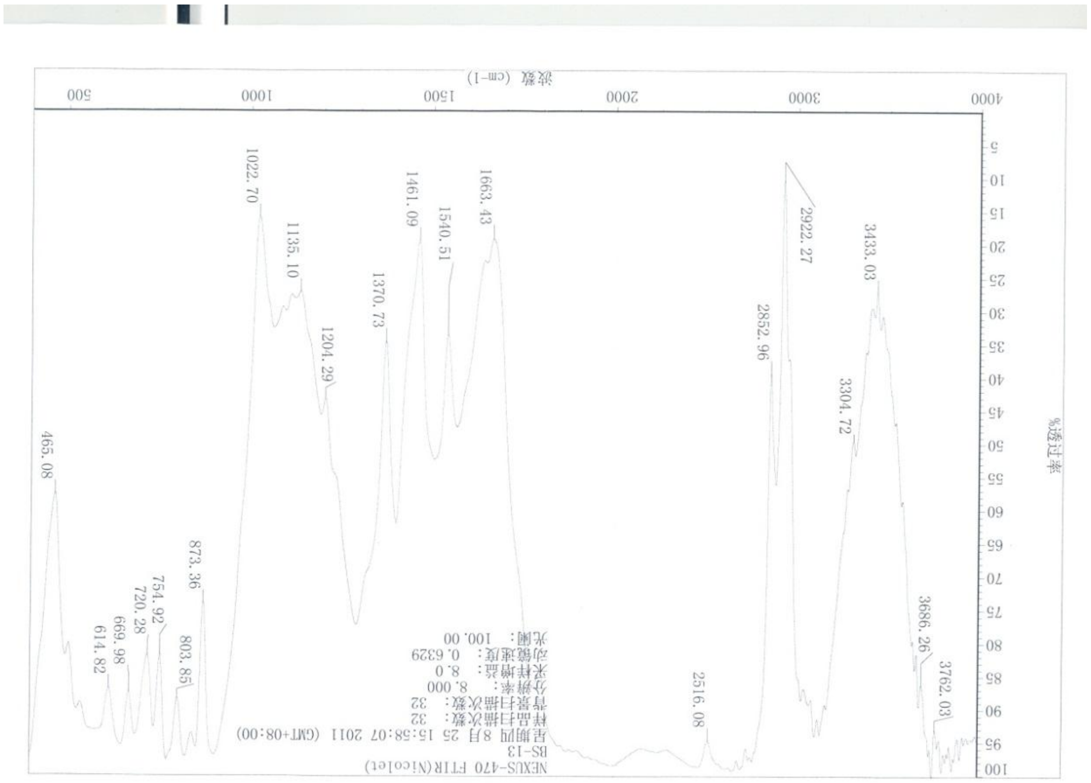

Fig 2-8 IR spectrum of **2**

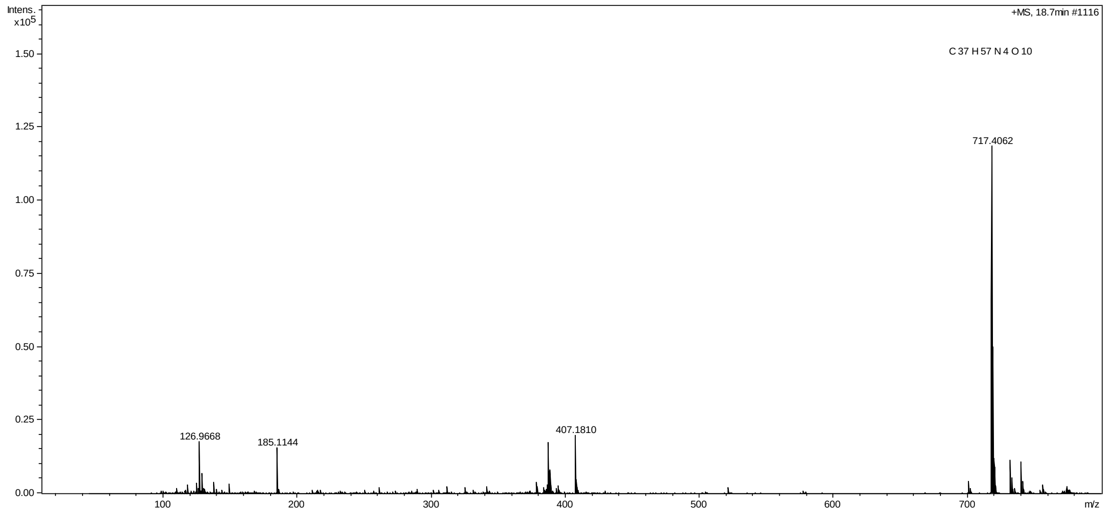

Fig 2-9 HRESIMS spectrum of **2**

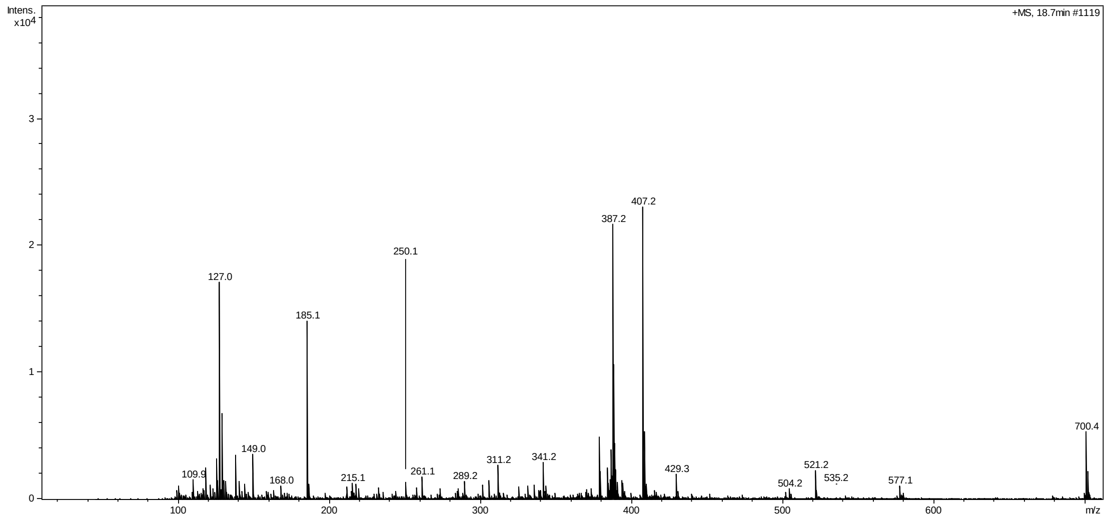

Fig 2-10 ESIMS spectrum of **2**

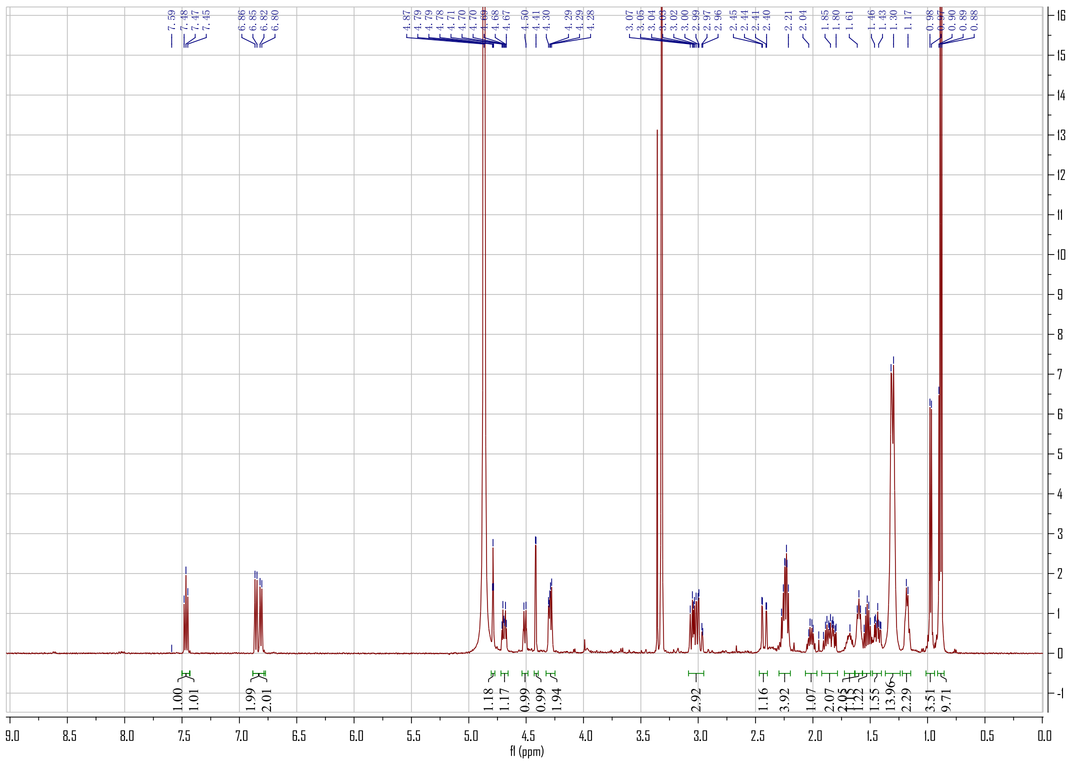

Fig 3-1 ^{1}HNMR spectrum of **3**

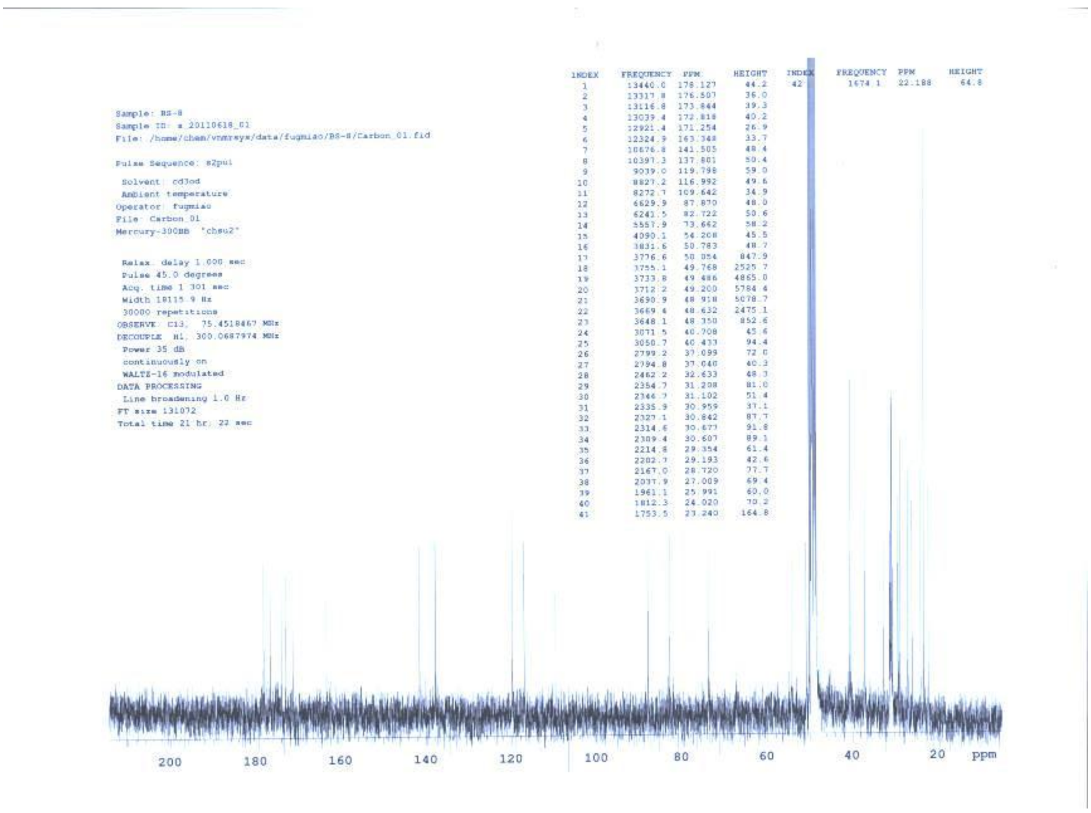

Fig 3-2 ^{13}CNMR spectrum of **3**

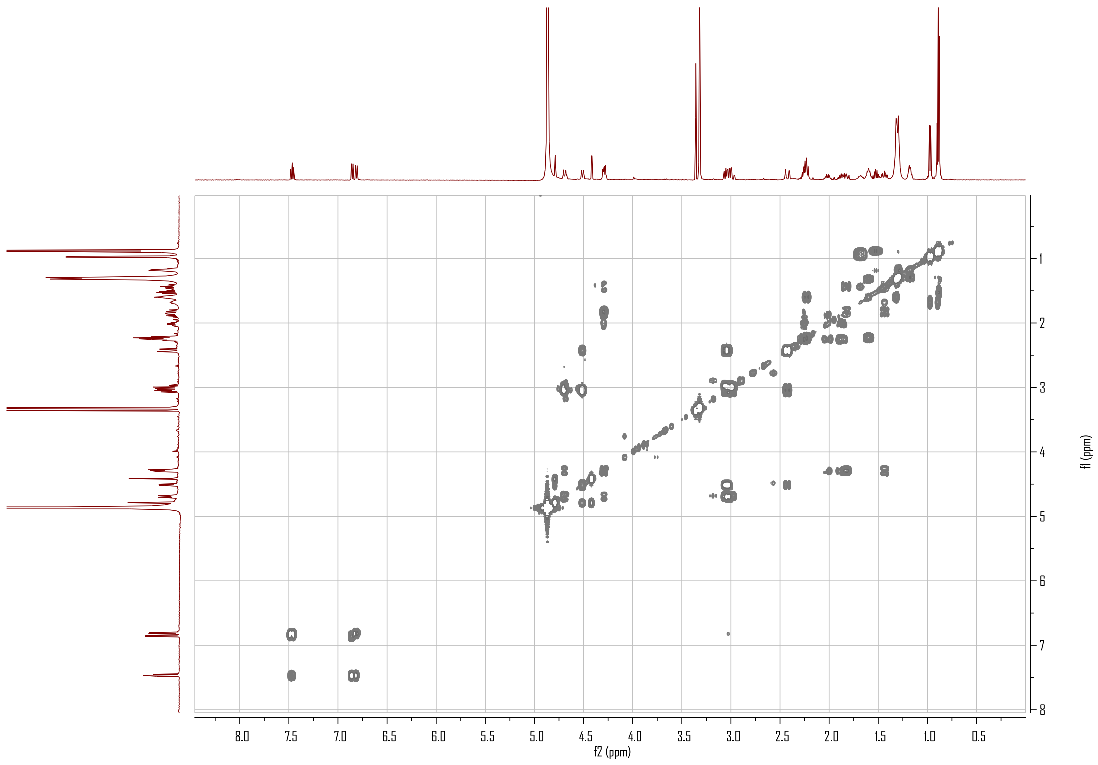

Fig 3-3 ^{1}H-^{1}HCOSY spectrum of **3**

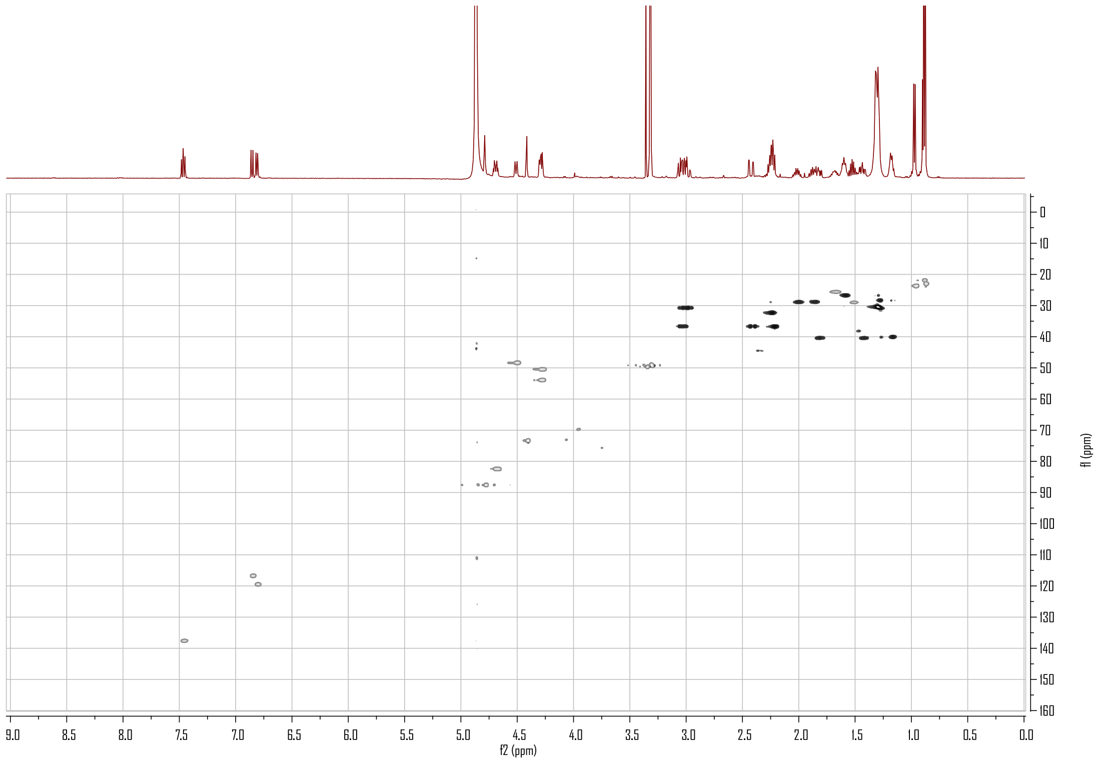

Fig 3-4 HMQC spectrum of **3**

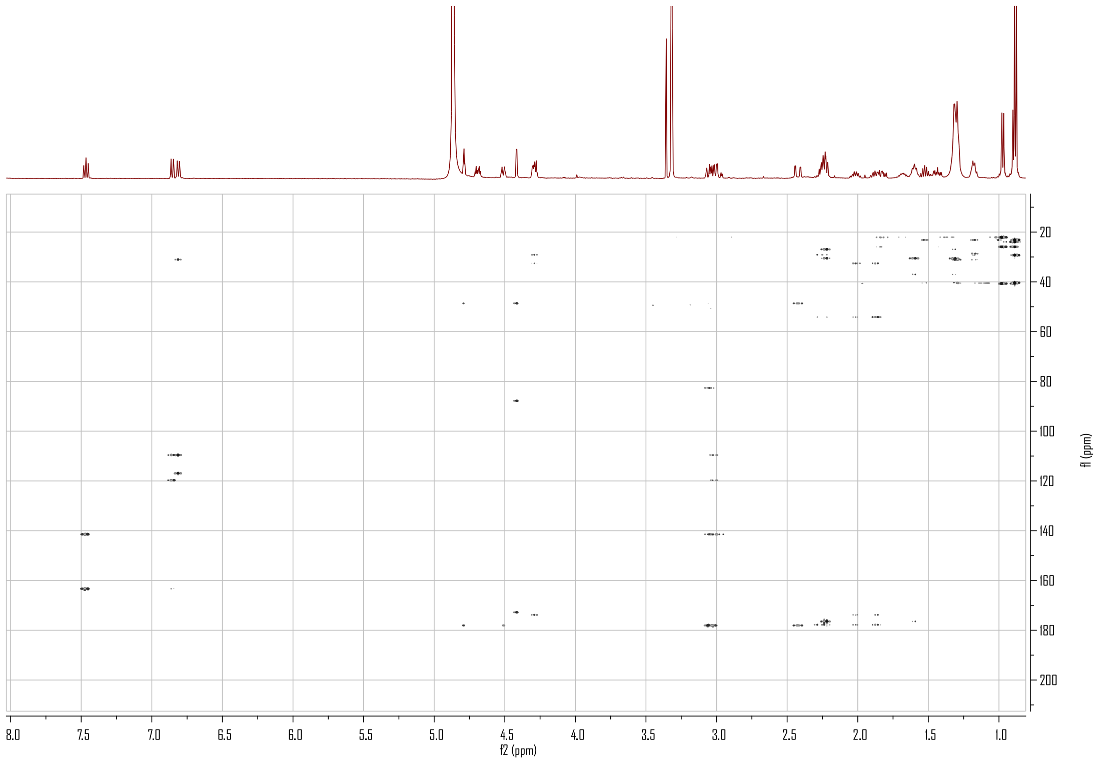

Fig 3-5 HMBC spectrum of **3**

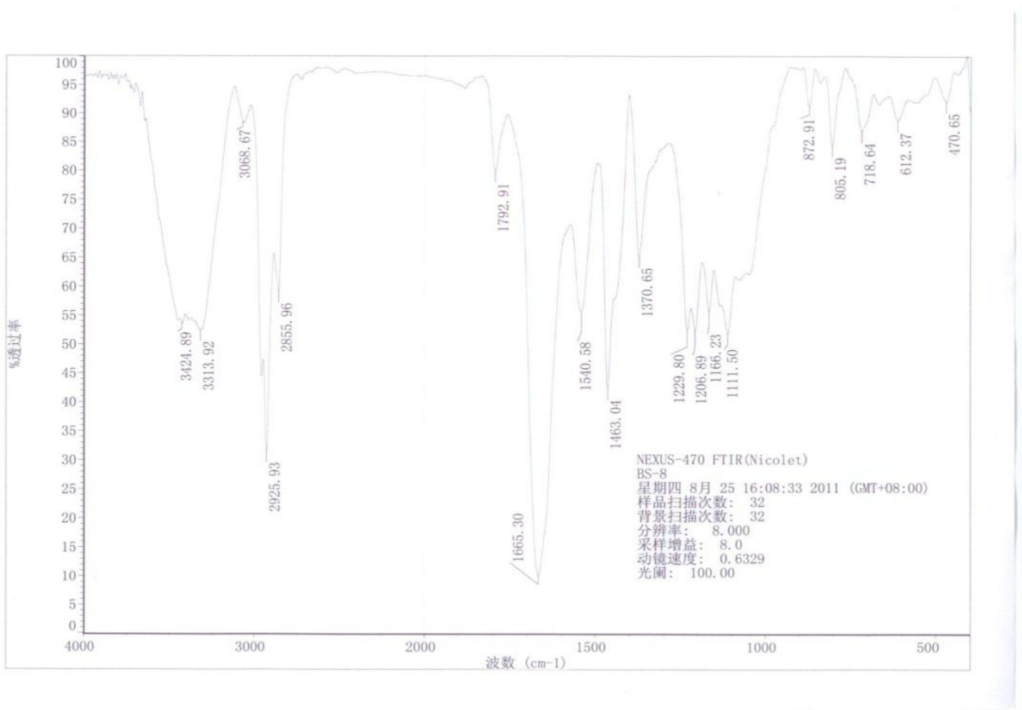

Fig 3-6 IR spectrum of **3**

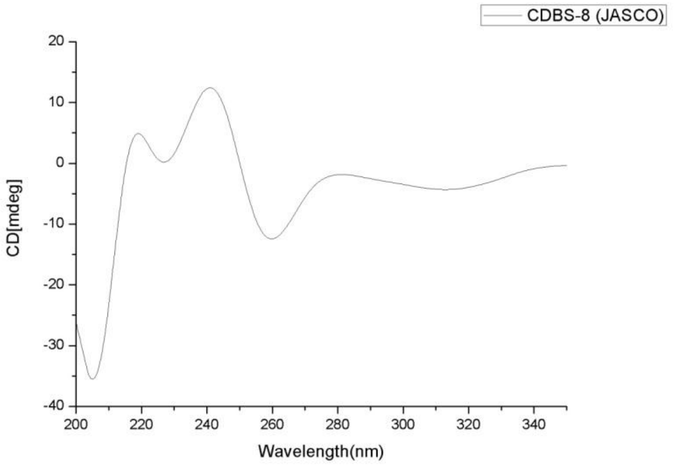

Fig 3-7 CD spectrum of **3**

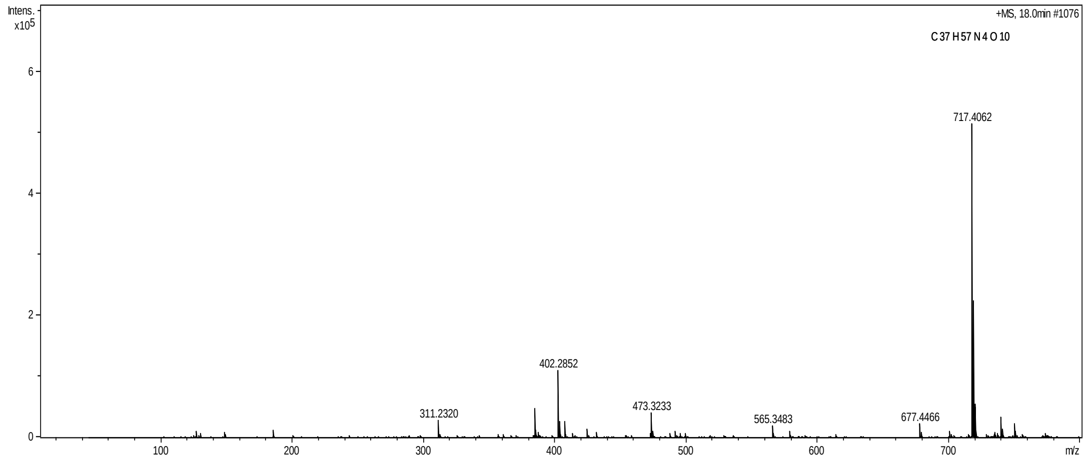

Fig 3-8 HRESIMS spectrum of **3**

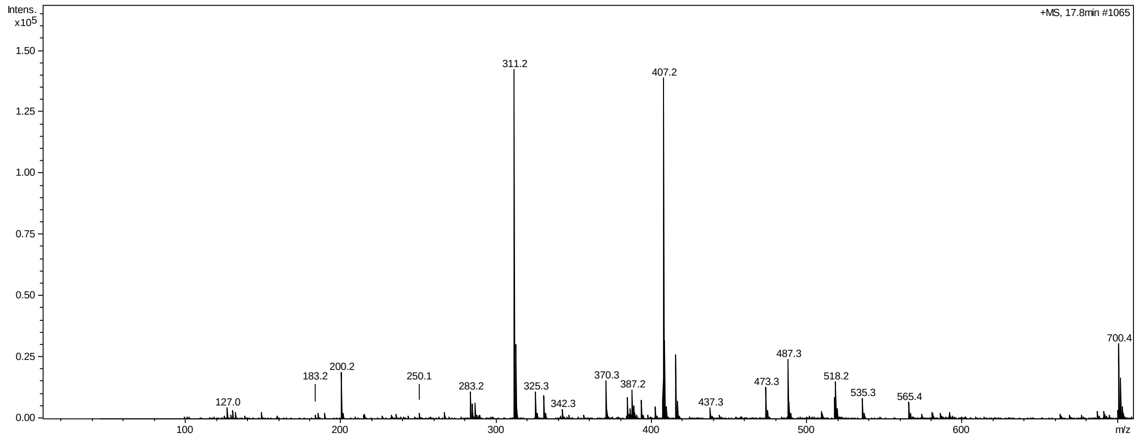

Fig 3-9 ESIMS spectrum of **3**

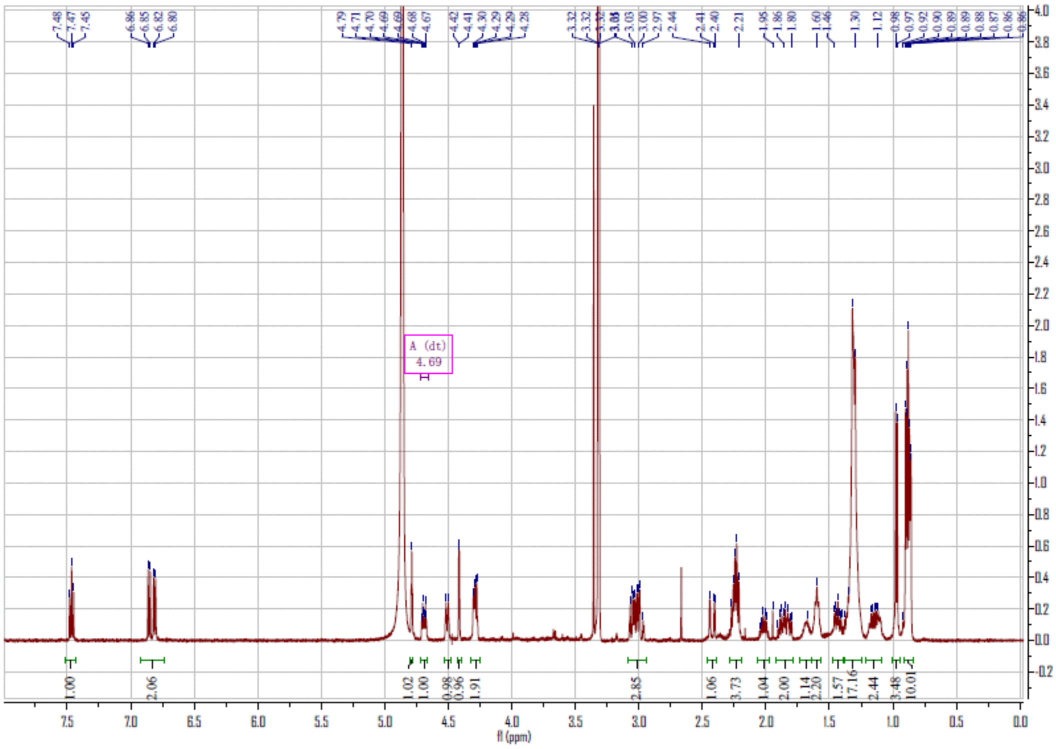

Fig 4-1 ^{1}HNMR spectrum of **4**

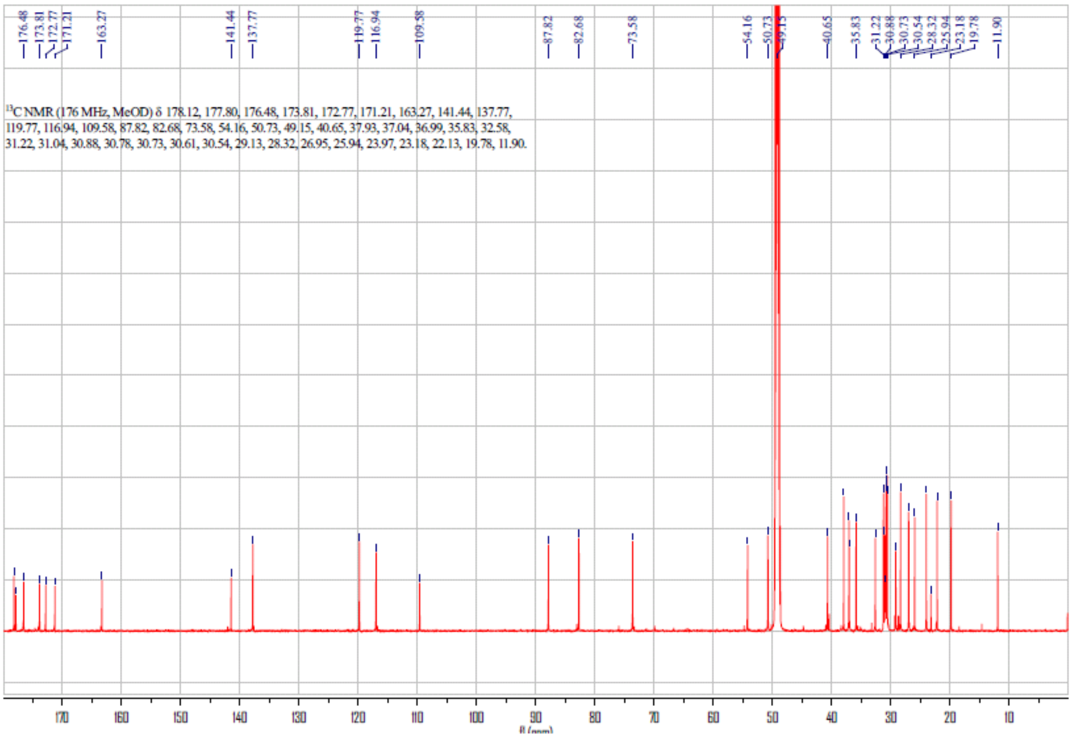

Fig 4-2 ^{13}CNMR spectrum of **4**

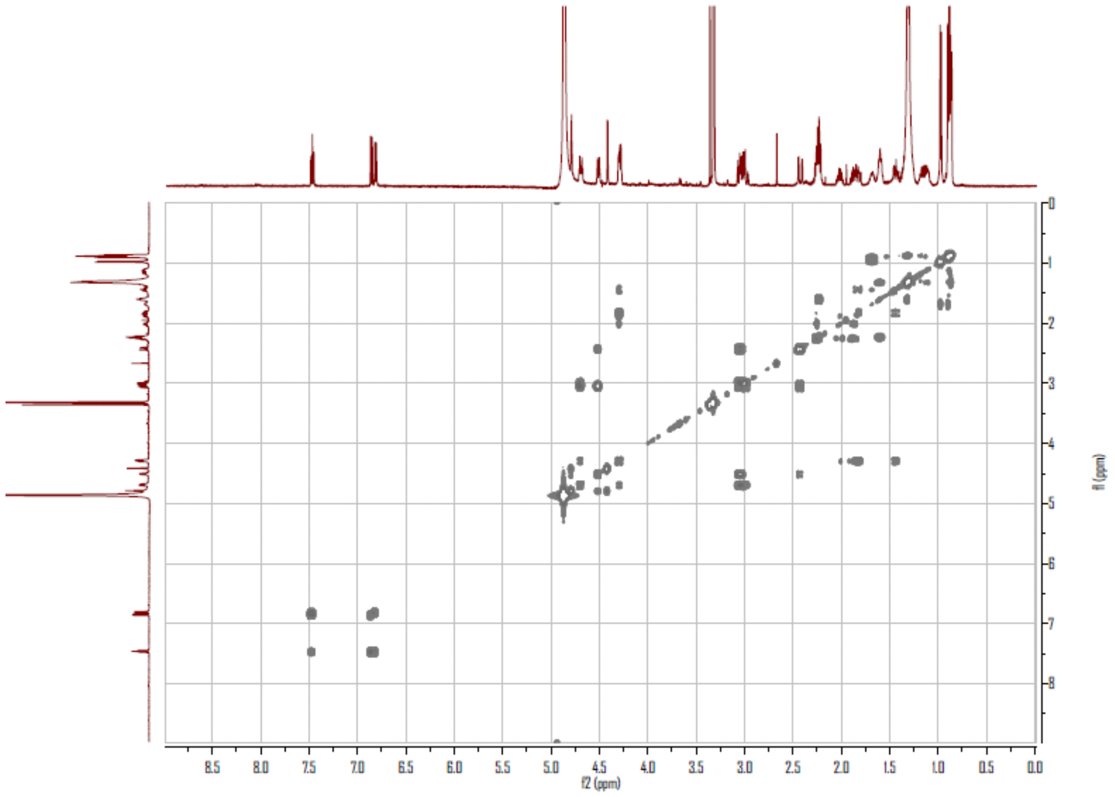

Fig 4-3 ^{1}H-^{1}HCOSY spectrum of **4**

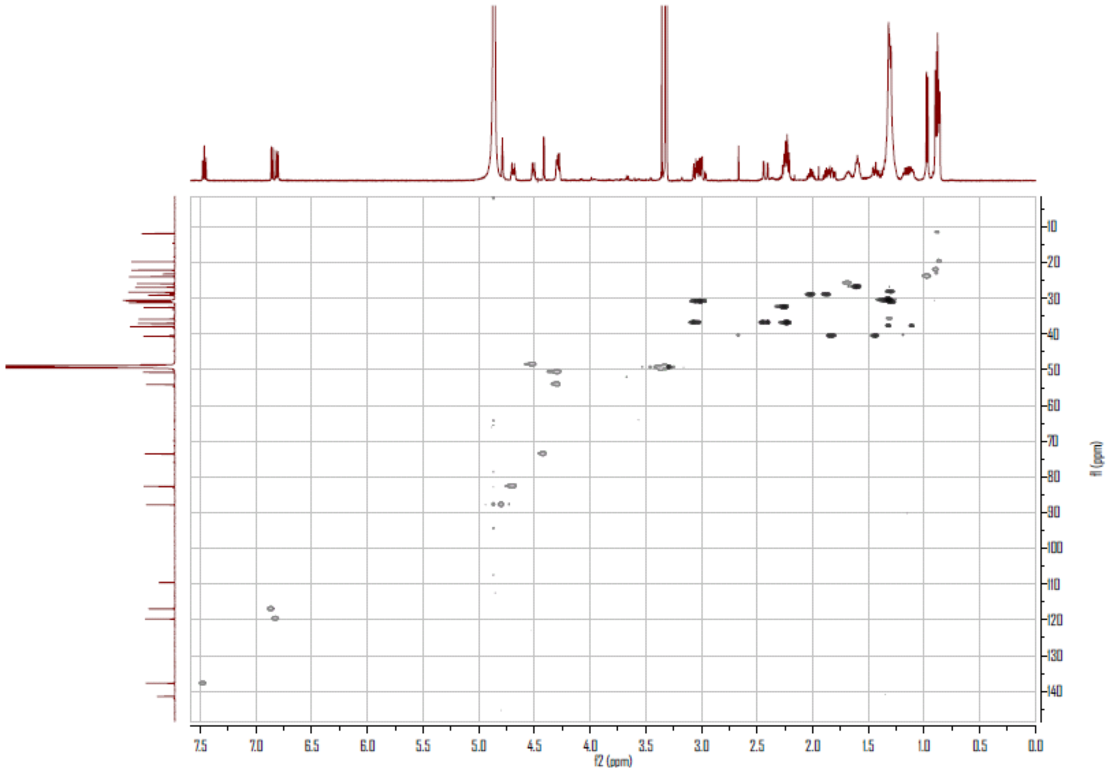

Fig 4-4 HMQC spectrum of **4**

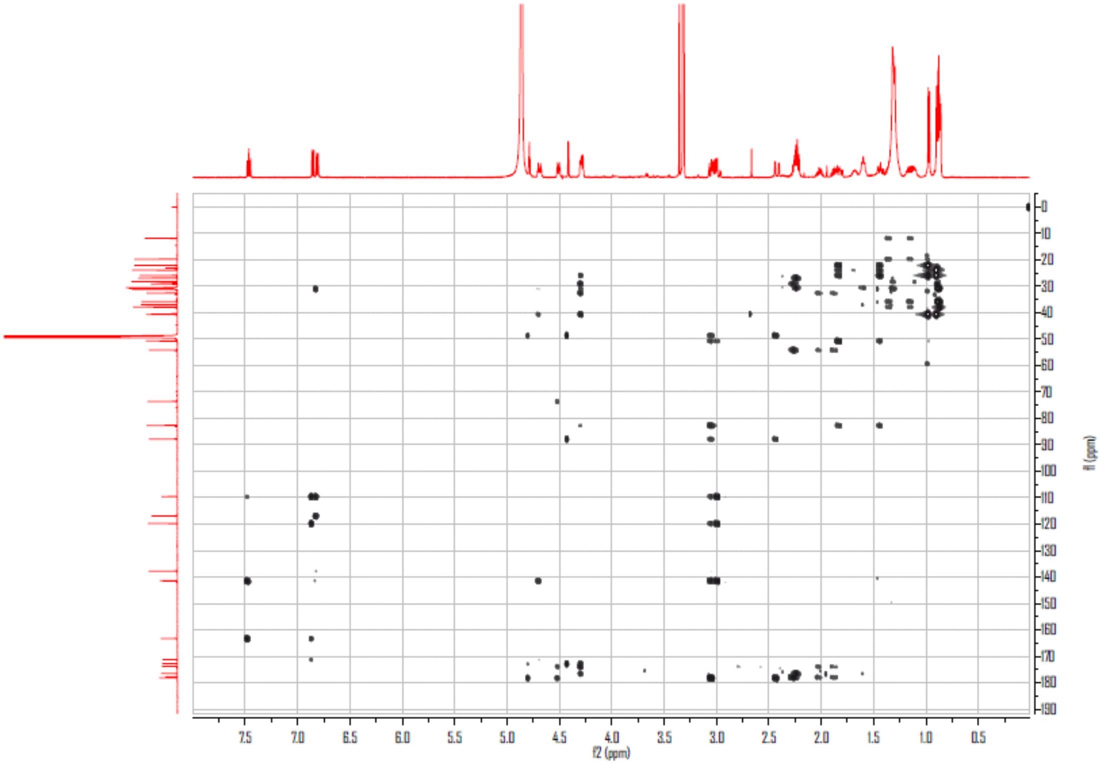

Fig 4-5 HMBC spectrum of **4**

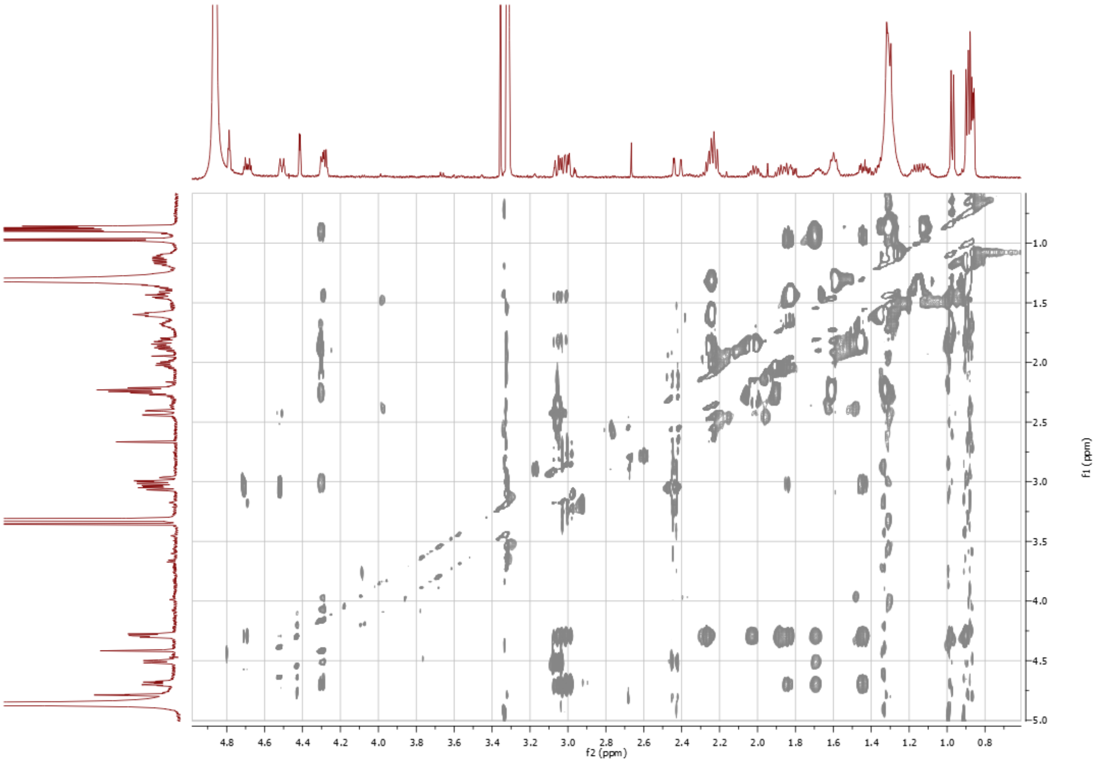

Fig 4-6 NOESY spectrum of **4**

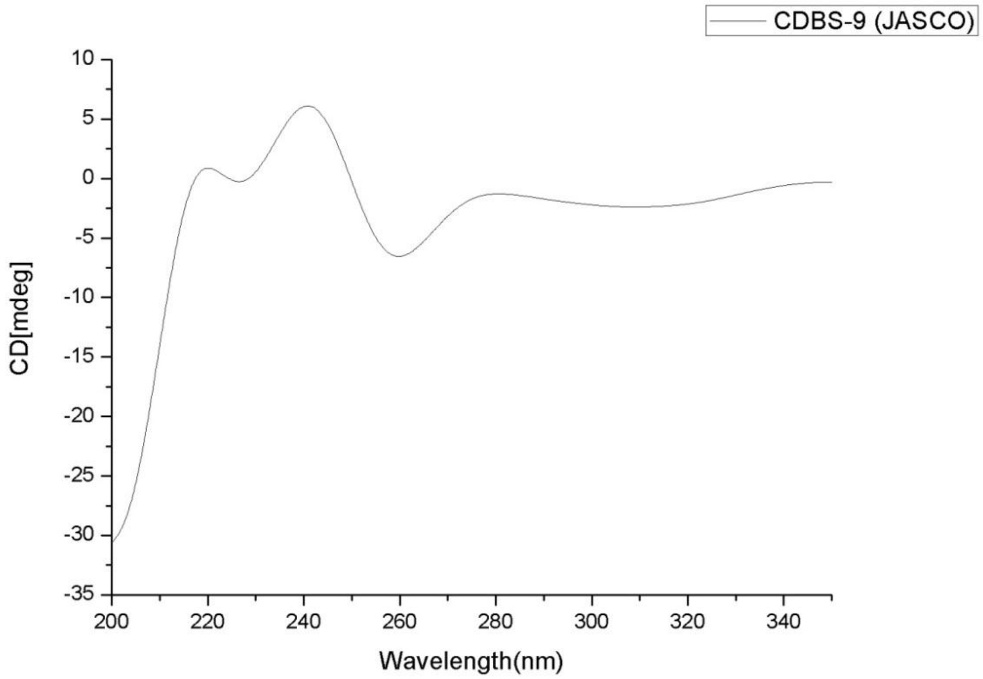

Fig 4-7 CD spectrum of **4**

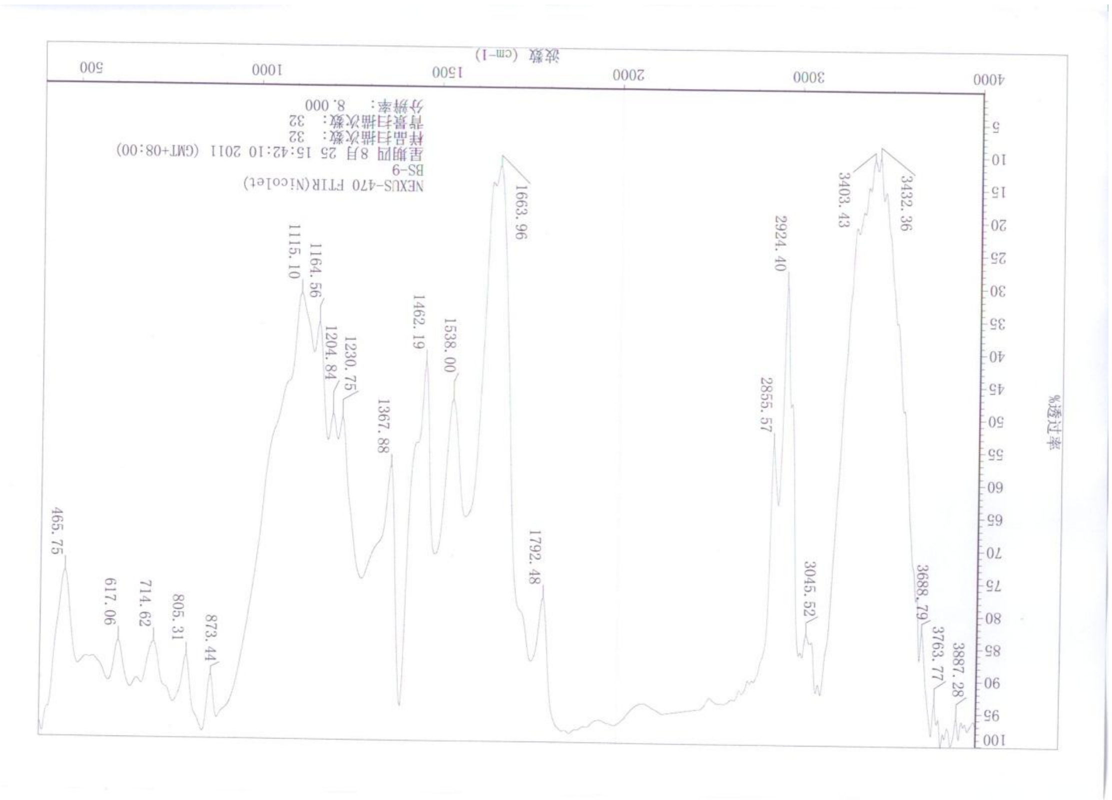

Fig 4-8 IR spectrum of **4**

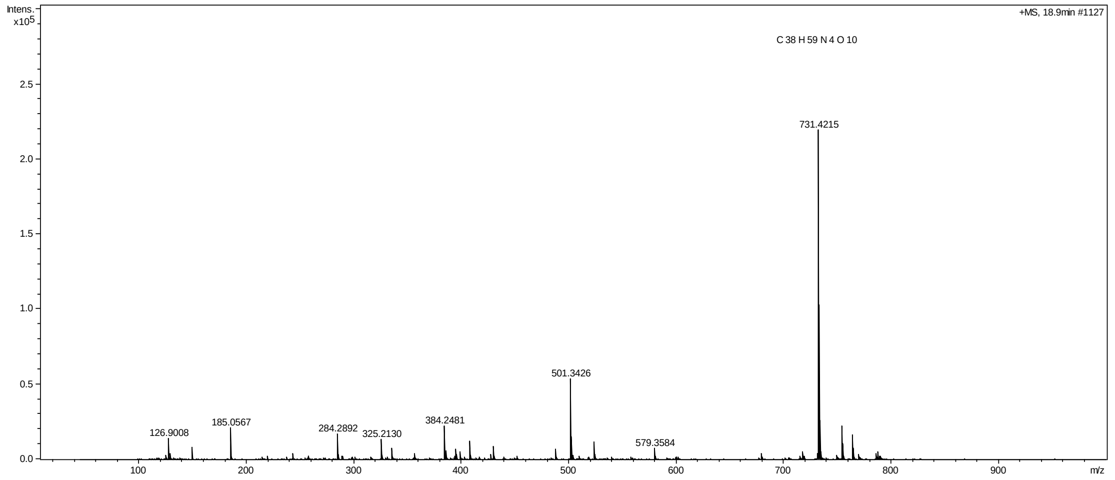

Fig 4-9 HRESIMS spectrum of **4**

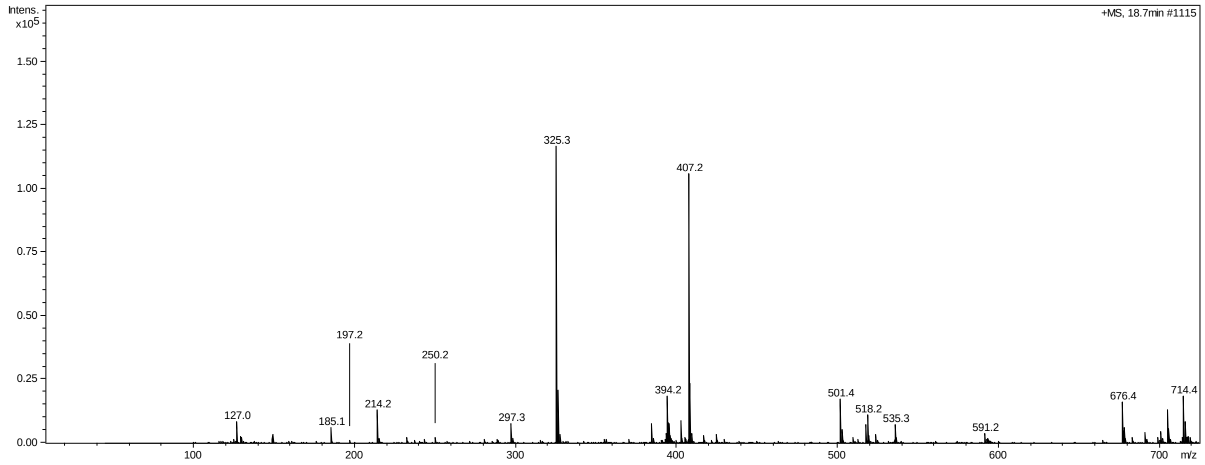

Fig 4-10 ESIMS spectrum of **4**

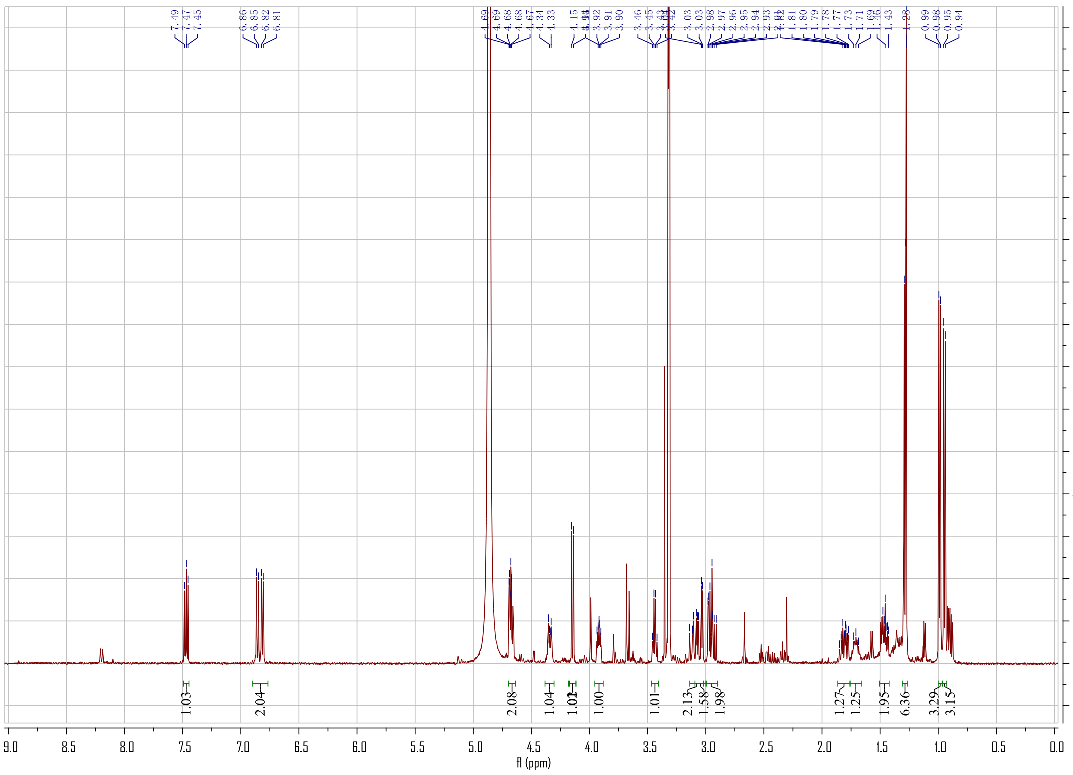

Fig 5-1 ^{1}HNMR spectrum of **5**

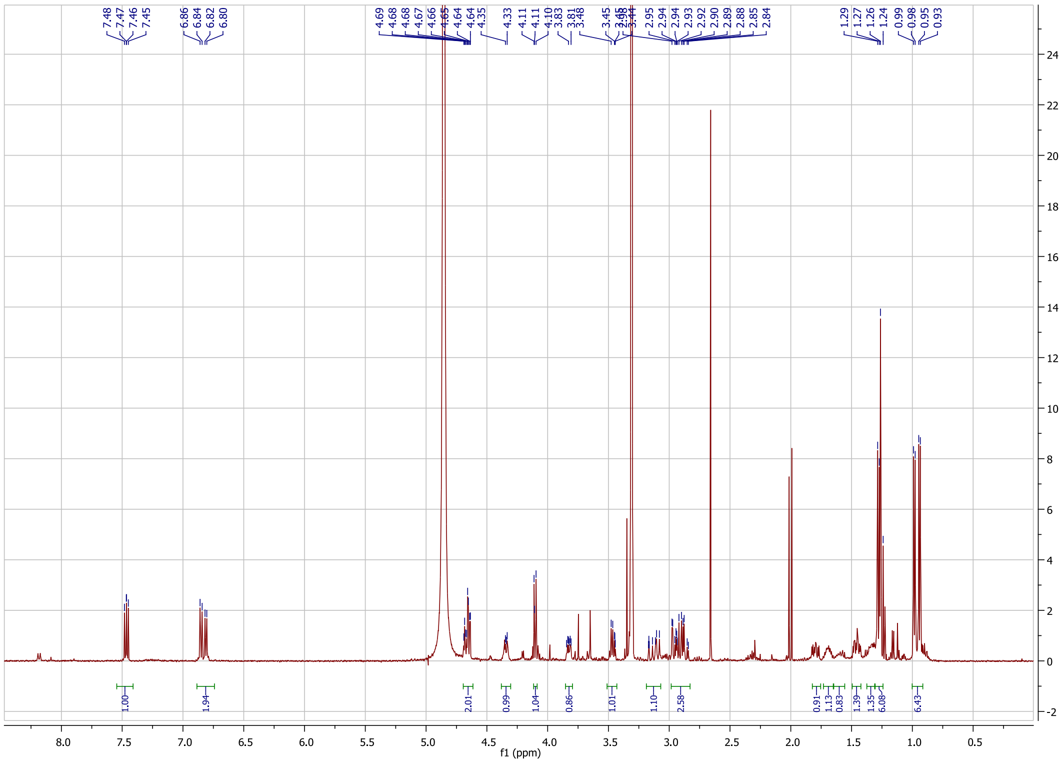

Fig 5-2 ^{1}HNMR spectrum of **6**

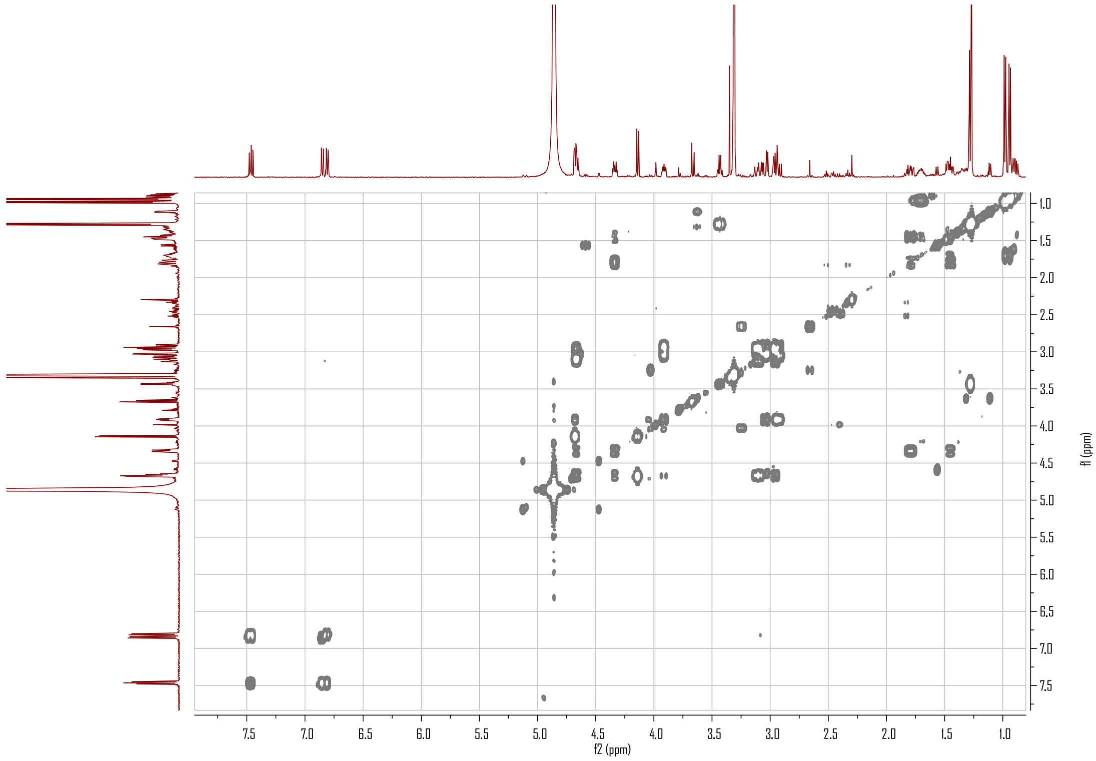

Fig 5-3 ^{1}H-^{1}HCOSY spectrum of **5**

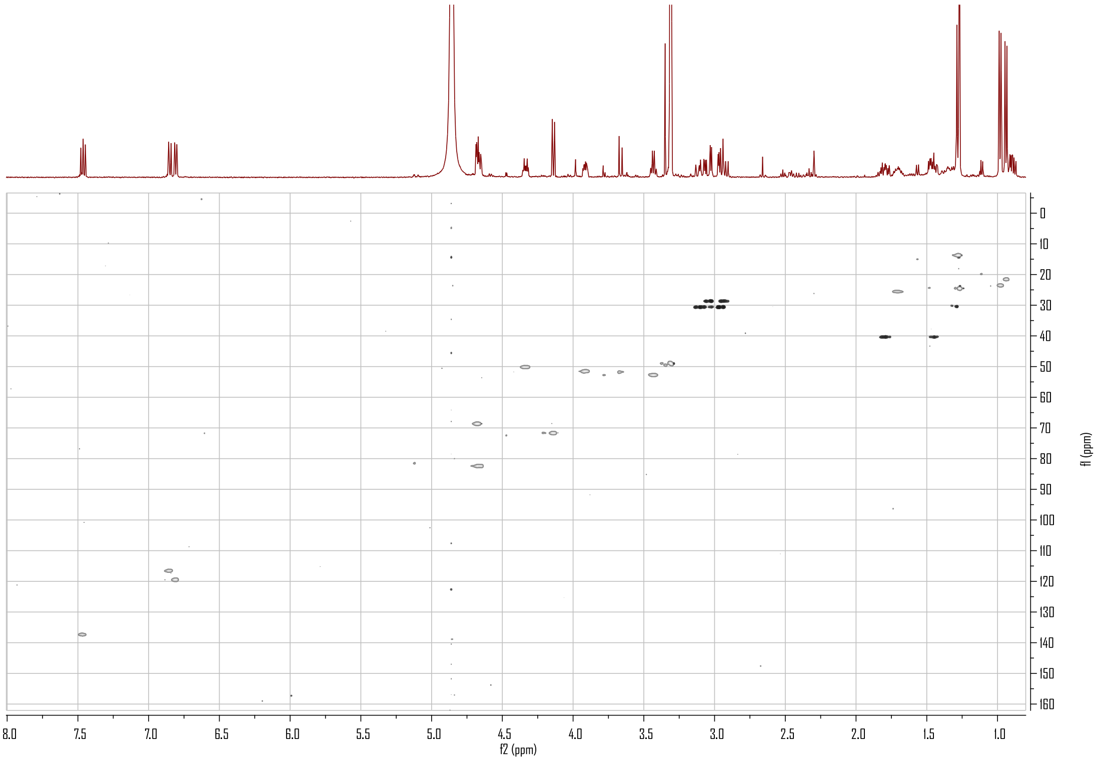

Fig 5-4 HMQC spectrum of **5**

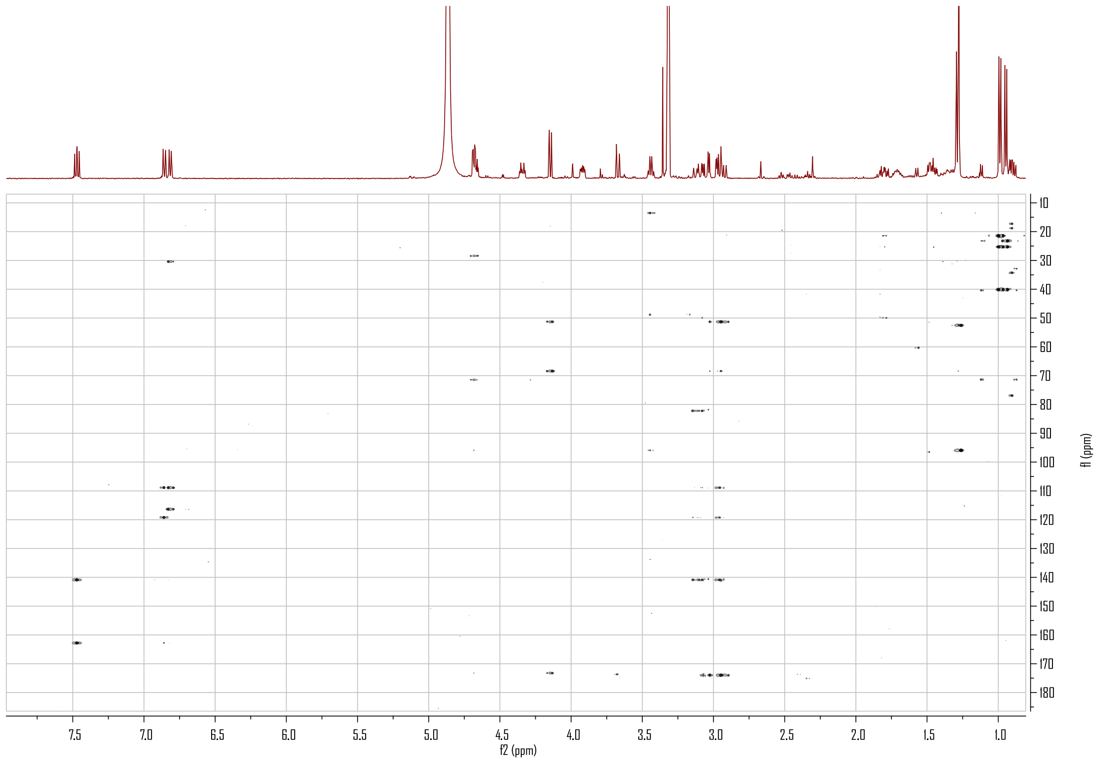

Fig 5-5 HMBC spectrum of **5**

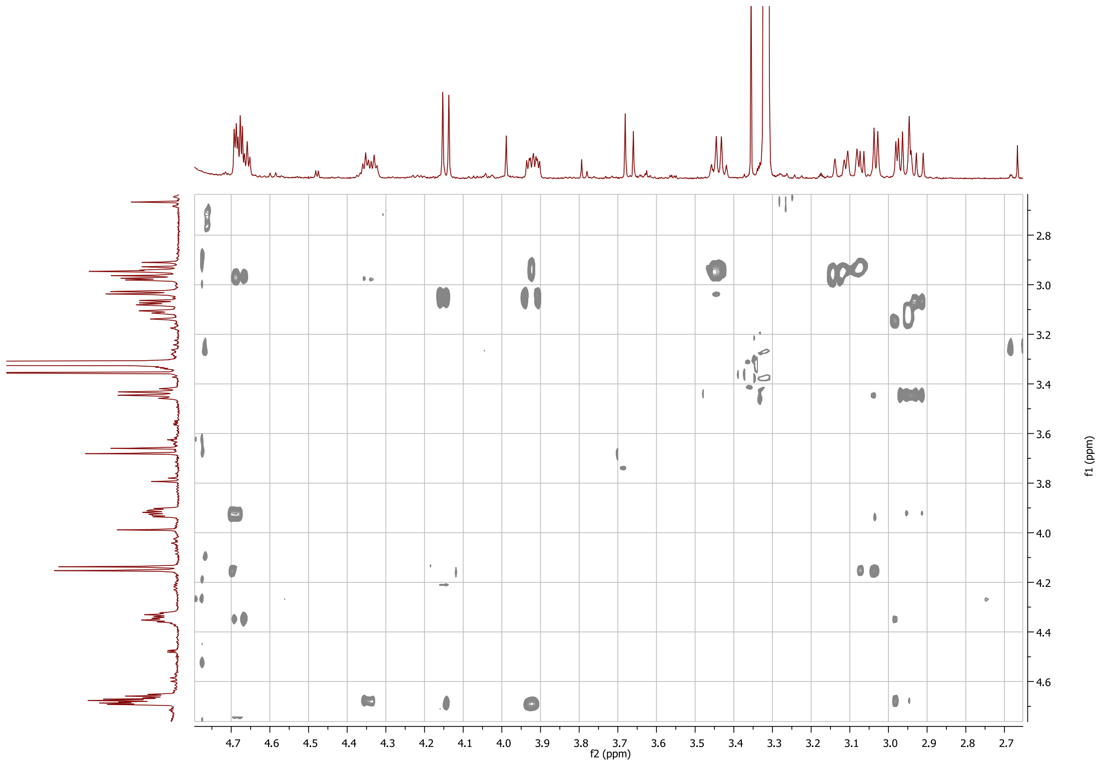

Fig 5-6 NOESY spectrum of **5**

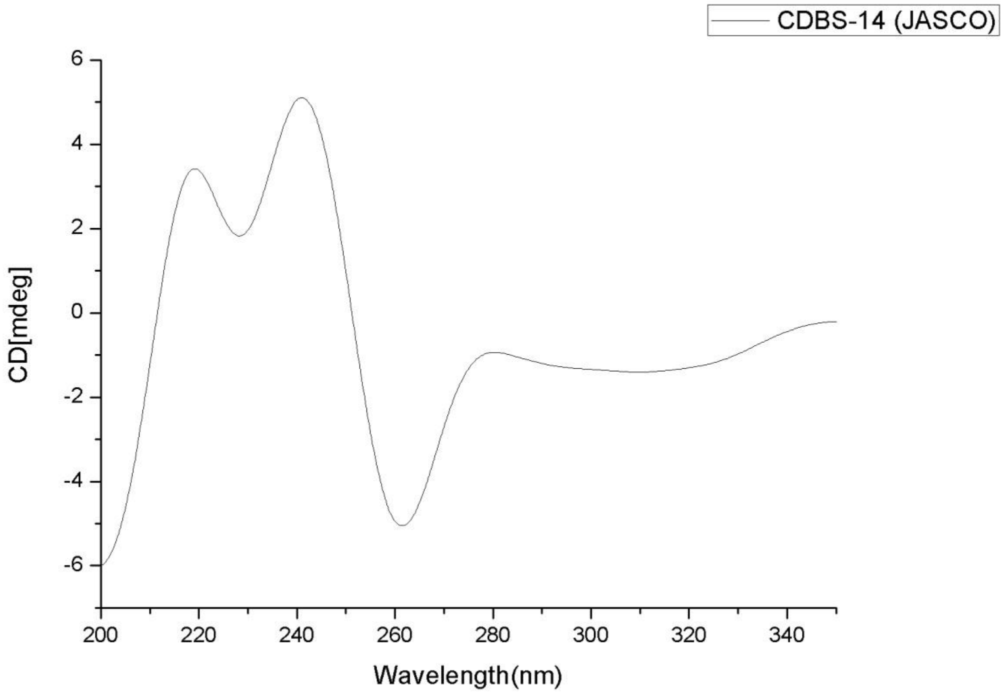

Fig 5-7 CD spectrum of **5**

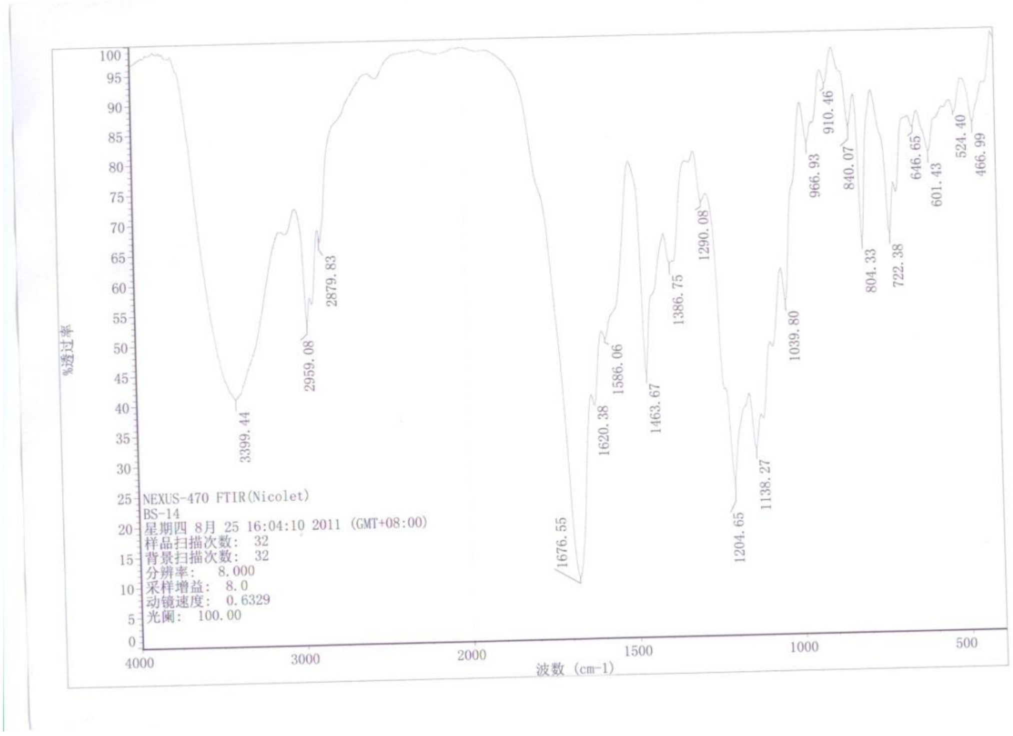

Fig 5-8 IR spectrum of **5**

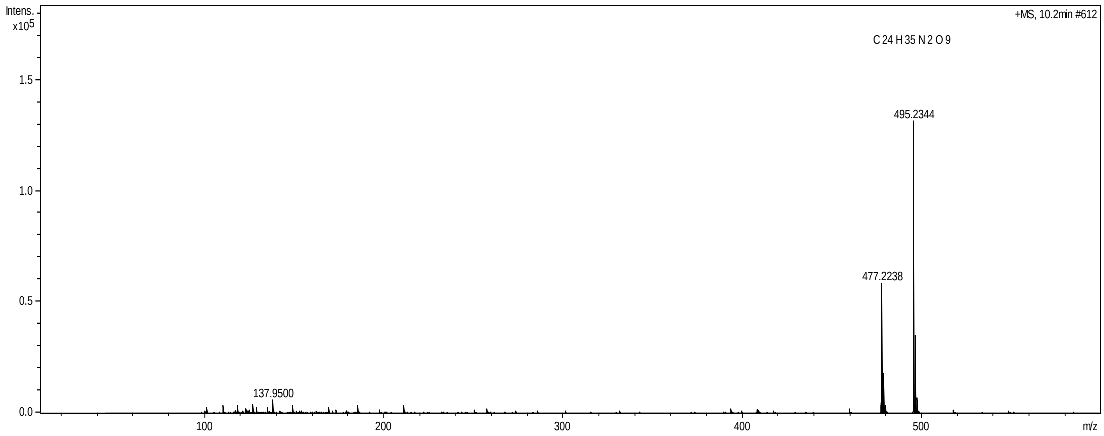

Fig 5-9 HRESIMS spectrum of **5**
